# Humoral Immunogenicity of mRNA-1345 RSV Vaccine in Older Adults

**DOI:** 10.1093/infdis/jiae316

**Published:** 2024-06-18

**Authors:** Jaya Goswami, Abdullah H Baqui, Pablo A Doreski, Gonzalo Perez Marc, Gilberto Jimenez, Salahuddin Ahmed, Khalequz Zaman, Christopher J A Duncan, Mugen Ujiie, Mika Rämet, Lina Pérez–Breva, Lan Lan, Jiejun Du, Archana Kapoor, Shraddah Mehta, Joanne E Tomassini, Wenmei Huang, Honghong Zhou, Sonia K Stoszek, Frances Priddy, Nina Lin, Nancy Le Cam, Christine A Shaw, Karen Slobod, Eleanor Wilson, Jacqueline M Miller, Rituparna Das

**Affiliations:** Infectious Disease, Research and Development, Moderna, Inc., Cambridge, Massachusetts; Department of International Health, Johns Hopkins Bloomberg School of Public Health, Baltimore, Maryland; Hospital Militar Central Cirujano Mayor Dr. Cosme Argerich; Consultorios Médicos Dr. Doreski, Buenos Aires, Argentina; Spotlight Research Center, Miami, Florida; Department of International Health, Johns Hopkins University, Zakiganj, Sylhet, Bangladesh; Infectious Diseases Division, Matlab Health Research Center, Matlab Bazar, Bangladesh; Department of Infectious Diseases, Royal Victoria Infirmary, Newcastle upon Tyne, Northumberland, United Kingdom; Center for Global Health and Medicine, Shinjuku-Ku, Japan; Finnish Vaccine Research Ltd; Faculty of Medicine and Health Technology, Tampere University, Tampere, Finland; Vaccine Research, FISABIO–Public Health, Valencia, Spain; Infectious Disease, Research and Development, Moderna, Inc., Cambridge, Massachusetts; Infectious Disease, Research and Development, Moderna, Inc., Cambridge, Massachusetts; Infectious Disease, Research and Development, Moderna, Inc., Cambridge, Massachusetts; Infectious Disease, Research and Development, Moderna, Inc., Cambridge, Massachusetts; Infectious Disease, Research and Development, Moderna, Inc., Cambridge, Massachusetts; Infectious Disease, Research and Development, Moderna, Inc., Cambridge, Massachusetts; Infectious Disease, Research and Development, Moderna, Inc., Cambridge, Massachusetts; Infectious Disease, Research and Development, Moderna, Inc., Cambridge, Massachusetts; Infectious Disease, Research and Development, Moderna, Inc., Cambridge, Massachusetts; Infectious Disease, Research and Development, Moderna, Inc., Cambridge, Massachusetts; Infectious Disease, Research and Development, Moderna, Inc., Cambridge, Massachusetts; Infectious Disease, Research and Development, Moderna, Inc., Cambridge, Massachusetts; Infectious Disease, Research and Development, Moderna, Inc., Cambridge, Massachusetts; Infectious Disease, Research and Development, Moderna, Inc., Cambridge, Massachusetts; Infectious Disease, Research and Development, Moderna, Inc., Cambridge, Massachusetts; Infectious Disease, Research and Development, Moderna, Inc., Cambridge, Massachusetts

**Keywords:** RSV, immunogenicity, mRNA-1345, neutralizing antibody, binding antibody

## Abstract

**Background:**

The mRNA-1345 vaccine demonstrated efficacy against respiratory syncytial virus (RSV) disease with acceptable safety in adults aged ≥60 years in the ConquerRSV trial. Here, humoral immunogenicity results from the trial are presented.

**Methods:**

This phase 2/3 trial randomly assigned adults (≥60 years) to mRNA-1345 50-µg encoding prefusion F (preF) glycoprotein (n = 17 793) vaccine or placebo (n = 17 748). RSV-A and RSV-B neutralizing antibody (nAb) and preF binding antibody (bAb) levels at baseline and day 29 postvaccination were assessed in a per-protocol immunogenicity subset (PPIS; mRNA-1345, n = 1515; placebo, n = 333).

**Results:**

Day 29 nAb geometric mean titers (GMTs) increased 8.4-fold against RSV-A and 5.1-fold against RSV-B from baseline. Seroresponses (4-fold rise from baseline) in the mRNA-1345 groups were 74.2% and 56.5% for RSV-A and RSV-B, respectively. Baseline GMTs were lower among participants who met the seroresponse criteria than those who did not. mRNA-1345 induced preF bAbs at day 29, with a pattern similar to nAbs. Day 29 antibody responses across demographic and risk subgroups were generally consistent with the overall PPIS.

**Conclusions:**

mRNA-1345 enhanced RSV-A and RSV-B nAbs and preF bAbs in adults (≥60 years) across various subgroups, including those at risk for severe disease, consistent with its demonstrated efficacy in the prevention of RSV disease.

**Clinical Trials Registration:**

NCT05127434.

## Teaser

mRNA-1345 vaccine enhanced levels of RSV-A and RSV-B neutralizing antibody and preF binding antibody compared with placebo in adults (≥60 years) across various subgroups, including those at risk for severe disease, consistent with its demonstrated efficacy in the prevention of RSV disease.

Respiratory syncytial virus (RSV) is an important etiological agent of acute respiratory disease (ARD), with symptoms ranging from mild upper respiratory to severe lower respiratory tract disease (LRTD) in children and adults [1, 2]. The risk of severe RSV disease is increased in older adults with advanced age, frailty, and underlying medical conditions such as cardiovascular or pulmonary diseases [2–6]. Annual incidences of RSV infections in the United States and Europe are estimated to be 3%–7% in healthy adults aged ≥65 years and 4%–10% in high-risk adults, a population associated with increased morbidity and mortality [6–8]. In 2015, an estimated worldwide 336 000 hospitalizations and 14 000 in-hospital deaths were attributed to RSV infection in older adults [9]; in 2019, an estimated 5.2 million cases of RSV infection in developed countries led to 470 000 hospitalizations and 33 000 in-hospital deaths [3]. The high disease burden of RSV infection in older adults highlights the importance of protective vaccines in this population [2, 7].

Natural RSV infections induce incomplete immunity as evidenced by recurrent infections that are generally mild but can be severe, especially in at-risk populations [10]. Immune correlates of protection have not been clearly defined for RSV infection; however, both neutralizing antibody (nAb) and cell-mediated immune responses are known to enhance protection to natural infection [10–12]. The protective role of nAbs in RSV disease was demonstrated in studies showing that passive immunization of infants by maternal transfer and/or immunotherapy provided protection against RSV, including severe disease [13–15]. Low serum nAb levels have been shown to be associated with risk for severe disease [16]; in a small RSV challenge study, serum antibodies correlated with protection in older individuals [17]. Cell-mediated RSV-specific T-cell responses also play a role in protection against severe RSV-associated disease, and studies have shown that impaired CD4^+^ and CD8^+^ T-cell responses in older adults contribute to increased susceptibility to severe disease [5, 12, 18].

Current licensed and investigational RSV vaccines that target the stabilized prefusion (preF) conformation of the RSV fusion (F) glycoprotein have demonstrated high nAb responses against both RSV-A and RSV-B subtypes [11, 19–22] and efficacy in the prevention of RSV with acceptable safety [23–28]. Administration of mRNA-1345 to adults in a phase 1 study enhanced serum levels of nAbs against RSV-A and RSV-B that persisted through 12 months with an acceptable safety profile in older adults [29, 30]. A subsequent pivotal randomized, double-blind phase 2/3 trial (ConquerRSV) of mRNA-1345 conducted in >35 000 participants globally demonstrated the efficacy of mRNA-1345 against RSV-LRTD and RSV-ARD in adults ≥60 years of age, with no evidence of safety concerns during a median 3.7-month follow-up [28], that remained durable in a longer-term median follow-up of 8.6 months [31]. Here, we describe the immunogenicity results of that trial.

## METHODS

### Study Design and Participants

Humoral immunogenicity results at day 29 postvaccination from an ongoing randomized, double-blind, placebo-controlled, phase 2/3 study (ClinicalTrials.gov: NCT05127434) that evaluated a single dose of mRNA-1345 50 µg encoding the membrane-anchored RSV fusion glycoprotein (derived from RSV-A A2 strain) or placebo in adults aged ≥60 years are presented [28]. The trial was conducted in accordance with the International Council for Technical Requirements for Registration of Pharmaceuticals for Human Use, Good Clinical Practice Guidance, and applicable national, state, and local government regulations. The applicable central institutional review boards across 269 study sites in 22 countries approved the protocol and consent forms. All participants provided written informed consent. Details of the study design are provided in the protocol (Supplementary Materials).

Eligible participants were adults ≥60 years of age, including those with stable chronic medical conditions. Additional inclusion/exclusion criteria were previously reported [28] and are provided in the Supplementary Methods. Trial participants were randomly assigned 1:1 to mRNA-1345 or placebo using an interactive response technology system. Randomization was stratified by age (60–74 vs ≥75 years) and risk factors, including presence/absence of congestive heart failure (CHF) and/or chronic obstructive pulmonary disease (COPD). Participants with other high-risk stable medical conditions (asthma, chronic respiratory disease, diabetes, advanced liver disease, renal disease) were also included. The frailty of participants was assessed (Edmonton Frail Scale) [32] and classified as fit, vulnerable, or frail.

### Study Objectives

Results of the primary efficacy and safety objectives of the ConquerRSV trial have been previously reported; study objectives are detailed in the protocol and statistical analysis plan (Supplementary Materials) [28]. A secondary objective of the study, the immunogenicity of mRNA-1345, is to evaluate the immune response to a single dose of mRNA-1345 vaccine from baseline up to 24 months postinjection. Day 29 immunogenicity results are presented here. Day 15 results from a limited number of participants in phase 2 of the study are also provided. The immunogenicity endpoints described in this report include geometric mean titers (GMTs) of serum RSV nAbs, geometric mean concentrations (GMCs) of serum RSV preF binding antibodies (bAbs) at baseline (day 1) before study injection and at day 29, seroresponse rates (SRRs) of serum RSV nAbs and bAbs, geometric mean fold rises (GMFRs) of postinjection/baseline titers for RSV nAbs and bAbs, and proportions of participants with ≥2-fold increases in RSV nAb titers and bAb concentrations at day 29. Seroresponse for RSV nAbs was defined as a postinjection titer ≥4 times the lower limit of quantification (LLOQ) if baseline was <LLOQ or a ≥4-fold increase from baseline in postinjection titers if baseline was ≥LLOQ. Serum nAb and bAb titers at day 29 (and day 15, phase 2) were assessed using validated microneutralization and quantitative Luminex assays, respectively (Supplementary Methods).

### Statistical Analysis

**Figure 1.**
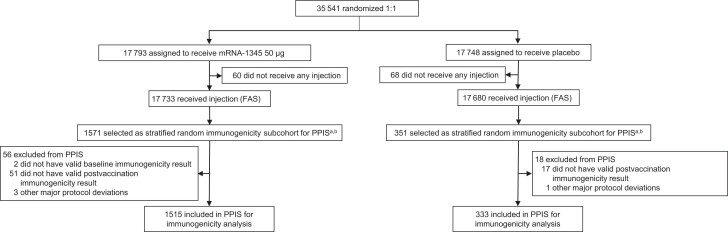
Summary of stratification for random immunogenicity subcohort (Supplementary Table 1). ^a^Random sampling stratified by age (60–74 y and ≥75 y), lower respiratory tract disease (LRTD) risk factor (absent/present), and region (Northern/Southern Hemisphere). ^b^Per-protocol immunogenicity set: participants in the random immunogenicity subcohort who received the assigned vaccine dose according to protocol and had respiratory syncytial virus (RSV) immunogenicity titer results at baseline, ≥1 valid result after vaccine administration, and no major protocol deviations affecting the primary immunogenicity outcomes; participants identified with an event of RSV acute respiratory disease or RSV-LRTD postbaseline were not excluded. Abbreviations: FAS, full analysis set; PPIS, per-protocol immunogenicity set.

To characterize immunogenicity, a stratified random sample of participants in the study (random immunogenicity subcohort) was selected from all participants randomly assigned in the study who received study vaccine by 31 October 2022 (Supplementary Methods, Figure 1, Supplementary Table 1). Participants were randomized in a 5:1 ratio of mRNA-1345 to placebo and stratification was based on age (60–74 and ≥75 years), LRTD risk factor (absent/present), and region (Northern/Southern Hemisphere) to ensure that groups at high risk of severe RSV disease, such as the elderly and those with LRTD risk factors, were adequately represented.

Immunogenicity was assessed in the per-protocol immunogenicity subset (PPIS), consisting of participants in the random immunogenicity subcohort who received the assigned study vaccine and had RSV immunogenicity data at baseline (day 1, prevaccination) and ≥1 valid result postvaccination at day 29 (and day 15, phase 2) with no major protocol deviations affecting the primary immunogenicity outcomes (Supplementary Methods, Supplementary Table 1). Participants were analyzed according to the vaccination group assigned at randomization. Additional details of the random sampling of the analysis population and immunogenicity analysis are provided in the Supplementary Methods.

Serum samples for the immunogenicity assessment were collected at baseline (day 1), day 29, and day 15 (phase 2). The GMTs for RSV-A and RSV-B nAbs, GMCs for RSV preF bAbs, and GMFRs (day 29 postinjection/baseline GMTs or GMCs) of RSV-A and RSV-B nAbs and preF bAbs with corresponding 95% confidence intervals (CIs) were determined, as well as median, minimum, and maximum GMT/GMC levels. The 95% CIs were calculated based on *t-*distributions of the log-transformed values, then back-transformed to the original scale for presentation. The SRRs (postinjection titers of ≥4× LLOQ if baseline was <LLOQ or ≥4-fold increase from baseline if baseline was ≥LLOQ) for RSV-A and RSV-B nAbs and preF bAbs at each postbaseline time point are provided, with 2-sided 95% CIs (Clopper-Pearson method) by treatment group. The proportions of participants with ≥2-fold increases from baseline in RSV-A and RSV-B nAb GMTs, and preF bAb GMCs at each postbaseline time point, are provided with 2-sided 95% CIs (Clopper-Pearson method) by treatment group. An analysis of baseline nAb GMTs among participants who met or did not meet the SRR criterion was also performed.

The data cutoff date for the day 29 (and day 15, phase 2) immunogenicity analysis was 30 April 2023. All analyses were conducted using SAS software version 9.4 or higher.

## RESULTS

### Trial Population

A total of 1922 randomly selected participants in the full analysis set were included in the random immunogenicity subcohort (Figure 1). Of these participants, 56 (3.6%) in the mRNA-1345 and 18 (5.1%) in the placebo groups were excluded from the PPIS, leaving 1515 (96.4%) and 333 (94.9%) participants in these groups, respectively. The most common reason for exclusion was lack of valid immunogenicity results after study vaccine administration in the mRNA-1345 (51 [3.2%]) and placebo (17 [4.8%]) groups.

The baseline characteristics and demographics of participants in the PPIS in the mRNA-1345 and placebo groups were generally well-balanced (Table 1). The mean ages were 71.9 (range, 60–94) years in the mRNA-1345 group and 72.9 (range, 60–94) years in the placebo group, with 45.7% of participants ≥75 years and 15.3% of participants ≥80 years across both groups. In the overall PPIS, 45.1% of the participants were female, 77.9% were White, 8.7% were Black or African American, and 4.2% were Asian. In the mRNA-1345 and placebo groups, respectively, 38.6% and 45.0% of participants had underlying risk factors for LRTD and 57.2% and 58.6% had comorbidities at baseline, known to be associated with an increased risk of severe disease.

**Table 1.** Baseline Demographics and Characteristics of the Per-Protocol Immunogenicity Set

Characteristic	mRNA-1345, 50 µg (N = 1515)	Placebo (N = 333)	Total (N = 1848)
Age at enrollment, y			
Mean (range)	71.9 (60–94)	72.9 (60–94)	72.1 (60–94)
Age group^a^			
60–74 y	836 (55.2)	168 (50.5)	1004 (54.3)
≥75 y	679 (44.8)	165 (49.5)	844 (45.7)
Age by decade^a^			
60–69 y	619 (40.9)	120 (36.0)	739 (40.0)
70–79 y	673 (44.4)	154 (46.2)	827 (44.8)
≥80 y	223 (14.7)	59 (17.7)	282 (15.3)
LRTD risk factors^a^			
Present	585 (38.6)	150 (45.0)	735 (39.8)
CHF	110 (7.3)	35 (10.5)	145 (7.8)
COPD	444 (29.3)	108 (32.4)	552 (29.9)
CHF and COPD	31 (2.0)	7 (2.1)	38 (2.1)
Absent	930 (61.4)	183 (55.0)	1113 (60.2)
Sex			
Male	833 (55.0)	182 (54.7)	1015 (54.9)
Female	682 (45.0)	151 (45.3)	833 (45.1)
Race group			
White	1172 (77.4)	267 (80.2)	1439 (77.9)
Black or African American	133 (8.8)	27 (8.1)	160 (8.7)
Asian	66 (4.4)	12 (3.6)	78 (4.2)
Other, unknown, or not reported^b^	144 (9.5)	27 (8.1)	171 (9.3)
Ethnicity			
Hispanic or Latino	705 (46.5)	147 (44.1)	852 (46.1)
Not Hispanic or Latino	789 (52.1)	184 (55.3)	973 (52.7)
Unknown or not reported	21 (1.4)	2 (0.6)	23 (1.2)
World Bank Region			
North America/Europe	686 (45.3)	163 (48.9)	849 (45.9)
Central/Latin America/Africa	721 (47.6)	146 (43.8)	867 (46.9)
Asia Pacific	108 (7.1)	24 (7.2)	132 (7.1)
Edmonton Frail Scale			
0–3: Fit	1034 (68.3)	235 (70.6)	1269 (68.7)
4–5: Vulnerable	310 (20.5)	58 (17.4)	368 (19.9)
≥6: Frail	149 (9.8)	36 (10.8)	185 (10.0)
Missing	22 (1.5)	4 (1.2)	26 (1.4)
Comorbidities of interest^c^			
0	648 (42.8)	138 (41.4)	786 (42.5)
≥1	867 (57.2)	195 (58.6)	1062 (57.5)
BMI	n = 1512	n = 333	n = 1845
Mean BMI (SD), kg/m^2^	27.63 (4.21)	27.14 (4.35)	27.54 (4.24)

Data are presented as No. (%) unless otherwise indicated. Percentages are based on the number of participants in the per-protocol immunogenicity set. Percentages may not total 100% due to rounding. Data cutoff was 30 April 2023. Baseline value for Edmonton Frail Scale total score and BMI are defined as the most recent nonmissing measurement (scheduled or unscheduled) collected on or before the date of injection of mRNA-1345 or placebo.

Abbreviations: BMI, body mass index; CHF, congestive heart failure; COPD, chronic obstructive pulmonary disease; LRTD, lower respiratory tract disease; SD, standard deviation.

^a^Derived from age and risk factors collected on electronic case report forms. Assignment to vaccination groups was stratified by age (60–74 y vs ≥75 y) and risk factors for LRTD (present vs absent).

^b^Other race includes American Indian or Alaska Native, Native Hawaiian or other Pacific Islander, other, or multiple races.

^c^Comorbidities of interest include COPD, asthma, chronic respiratory disease, diabetes, CHF, advanced liver disease, or advanced renal disease.

### Immunogenicity

#### Neutralizing Antibody

**Figure 2.**
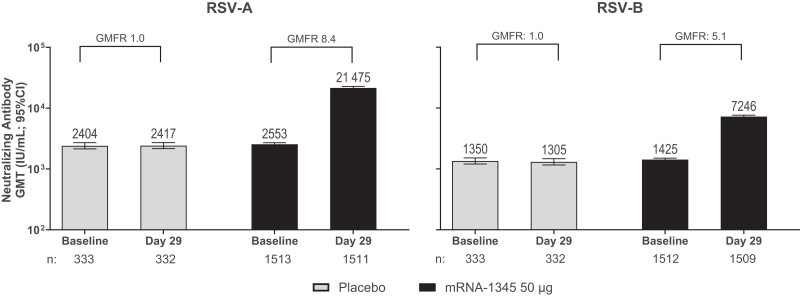
Neutralizing antibody geometric mean titers (GMTs) were assessed in the per-protocol immunogenicity set (PPIS). GMTs (IU/mL) of neutralizing antibodies for respiratory syncytial virus (RSV) subtypes A and B in participants in the PPIS were determined via microneutralization assays. Geometric mean fold rises (GMFRs) were determined for GMTs at day 29 vs baseline. The 95% confidence intervals (CIs) were calculated based on the *t*-distribution of the log-transformed values or the difference in the log-transformed values for geometric mean value and GMFR, respectively, then back-transformed to the original scale for presentation. Brackets represent GMFRs from baseline determined for GMTs at day 29 vs baseline; “n” indicates number of participants with nonmissing data at the visit (baseline or postbaseline).

Baseline nAb GMTs (95% CIs) against RSV-A subtype were similar for the mRNA-1345 (2552.8 [2414.3–2699.4]) and placebo (2403.7 [2136.0–2705.0]) groups (Table 2, Figure 2, Supplementary Figure 1). The GMTs (95% CIs) at day 29 against the RSV-A subtype increased 8.4-fold to 21 475.4 (20 273.9–22 748.1) from baseline following mRNA-1345 and were similar to baseline GMTs for placebo (2417.2 [2155.9–2710.0]). The GMTs (95% CIs) for RSV-B at baseline were also similar for mRNA-1345 (1425.4 [1352.7–1501.9]) and placebo (1350.3 [1203.3–1515.2]). At day 29, GMTs (95% CIs) increased 5.1-fold from baseline to 7246.0 (6864.8–7648.4) for mRNA-1345 and were similar to baseline levels for placebo (1304.7 [1160.0–1467.6]).

**Table 2.** Summary of Respiratory Syncytial Virus Subtypes A and B Neutralizing Antibody Titers by Visit in the Per-Protocol Immunogenicity Set by Microneutralization Assay

Visit	RSV-A		RSV-B	
	mRNA-1345, 50 µg (N = 1515)	Placebo (N = 333)	mRNA-1345, 50 µg (N = 1515)	Placebo (N = 333)
Day 1 (baseline), n	1513	333	1512	333
GMT, IU/mL	2552.8	2403.7	1425.4	1350.3
(95% CI)^a^	(2414.3–2699.4)	(2136.0–2705.0)	(1352.7–1501.9)	(1203.3–1515.2)
Min–max	175–259 061	157–106 190	94–112 476	114–79 619
Day 29, n	1511	332	1509	332
GMT, IU/mL	21 475.4	2417.2	7246.0	1304.7
(95% CI)^a^	(20 273.9–22 748.1)	(2155.9–2710.0)	(6864.8–7648.4)	(1160.0–1467.6)
Min–max	512–259 061	149–89 840	122–112 476	108–77 520
GM fold rise (95% CI)^a^	8.4 (8.0–8.9)	1.0 (1.0–1.1)	5.1 (4.9–5.4)	1.0 (0.9–1.0)
Seroresponse rate, n/N1 (%)^b^	1119/1509 (74.2)	2/332 (0.6)	851/1506 (56.5)	5/332 (1.5)
(95% CI)^c^	(71.9–76.3)	(0.1–2.2)	(54.0–59.0)	(0.5–3.5)
≥2-fold increase from baseline, n/N1 (%)^d^	1379/1509 (91.4)	15/332 (4.5)	1269/1506 (84.3)	18/332 (5.4)
(95% CI)^c^	(89.9–92.8)	(2.6–7.3)	(82.3–86.1)	(3.2–8.4)

GMTs (IU/mL) of neutralizing antibodies for RSV-A and RSV-B in participants in the per-protocol immunogenicity subset were determined in microneutralization assays. Antibody values reported as below the lower limit of quantification (LLOQ; 13 for RSV-A; 10 for RSV-B) are replaced by 0.5× LLOQ. Values greater than the upper limit of quantification (ULOQ; 259 061 for RSV-A; 112 476 for RSV-B) are replaced by the ULOQ.

Abbreviations: CI, confidence interval; GM, geometric mean; GMT, geometric mean titer; IU, international units; N, number of participants in the per-protocol immunogenicity set; n, number of participants with nonmissing data at the visit (baseline or postbaseline); N1, number of participants with nonmissing data at baseline and the corresponding postbaseline visit; RSV-A, respiratory syncytial virus subtype A; RSV-B, respiratory syncytial virus subtype B.

^a^95% CI was calculated based on the *t*-distribution of the log-transformed values or the difference in the log-transformed values for GM value and GM fold rise, respectively, then back-transformed to the original scale for presentation.

^b^Seroresponse at a participant level is defined as a change from below the LLOQ to ≥4× LLOQ, or at least a 4-fold increase if baseline is equal to or above the LLOQ. “n” is number of participants meeting the criterion at the time point. Percentages are based on N1.

^c^95% CIs were calculated using the Clopper-Pearson method.

^d^≥2-fold increase in GMT at day 29 from baseline at participant level is defined as a change from below the LLOQ to ≥2× LLOQ, or at least a 2-fold increase if baseline is equal to or above the LLOQ. Number of participants meeting the criterion at the time point. Percentages were based on N1.

The range of baseline titers were indicative of an adult population having had prior exposure to RSV-A and RSV-B subtypes. Day 29 nAb SRRs (95% CIs) for RSV-A were 74.2% (71.9%–76.3%) and 0.6% (0.1%–2.2%) for the mRNA-1345 and placebo groups, respectively; for RSV-B, SRRs were 56.5% (54.0%–59.0%) and 1.5% (0.5%–3.5%) for the corresponding groups. In an analysis of baseline GMTs among participants who met the SRR criteria (≥4-fold increase from baseline), nAb GMTs (95% CIs) against RSV-A were lower for those who met the criteria (2006.0 [1898.8–2119.3]) than those who did not (5062.5 [4469.6–5734.1]), with corresponding day 29 GMTs of 27 165.5 (25 581.2–28 847.8) and 10 978.8 (9755.0–12 356.1) and GMFRs (95% CIs) of 13.5 (12.9–14.2) and 2.2 (2.1–2.3) (Supplementary Table 2).

Baseline GMTs (95% CIs) against RSV-B were also lower in participants who met the SRR criteria (1038.7 [977.6–1103.6]) compared with those who did not (2142.1 [1975.6–2322.7]), with day 29 GMTs (95% CIs) of 10 197.7 (9544.9–10 895.2) and 4657.6 (4309.2–5034.2) and GMFRs (95% CIs) of 9.8 (9.4–10.3) and 2.2 (2.1–2.3). The proportions (95% CIs) of participants who achieved prespecified criterion for a ≥2-fold increase in GMTs from baseline in the mRNA-1345 groups were 91.4% (89.9%–92.8%) against RSV-A and 84.3% (82.3%–86.1%) against RSV-B. In the placebo group, 4.5% (2.6%–7.3%) and 5.4% (3.2%–8.4%) participants achieved a ≥2-fold increase in GMTs against RSV-A and RSV-B, respectively.

In a small group of phase 2 participants, the mRNA-1345 vaccine also increased nAb GMTs at day 15 for RSV-A and RSV-B (mRNA-1345, n = 68; placebo, n = 20), with GMFRs (95% CIs) of 9.2 (7.0–12.1) and 6.4 (4.9–8.2), respectively (Supplementary Table 3). The SRRs and proportions of participants who achieved ≥2-fold increases in GMTs from baseline were also consistent with day 29 responses.

**Figure 3.**
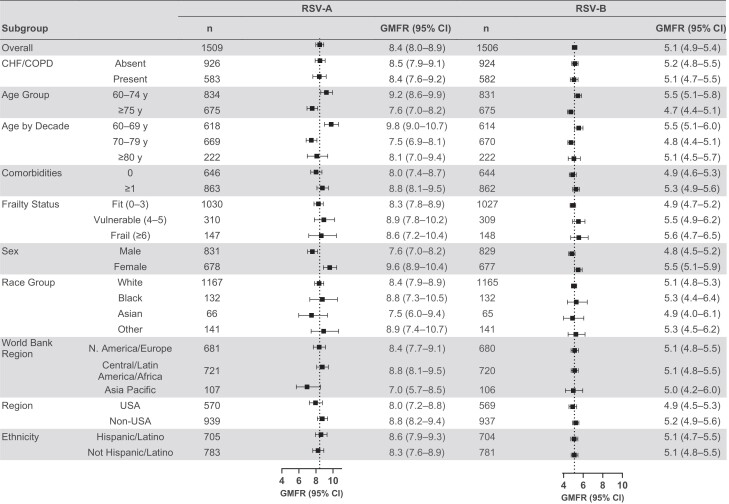
Neutralizing antibody geometric mean titers (GMTs) were assessed in the per-protocol immunogenicity set. Geometric mean fold rises (GMFRs) were determined for GMTs at day 29 vs baseline. The 95% confidence intervals (CIs) were calculated based on the *t*-distribution of the log-transformed values or the difference in the log-transformed values for geometric mean concentration and GMFR, respectively, then back-transformed to the original scale for presentation. Dotted lines represent the overall GMFR for respiratory syncytial virus (RSV) subtypes A and B; “n” indicates number of participants with nonmissing data at the visit (baseline or postbaseline). Age and risk factors were collected on electronic case report forms. Baseline values for Edmonton Frail Scale total score were defined as the most recent nonmissing measurement (scheduled or unscheduled) collected on or before the date of injection of mRNA-1345 or placebo. “Other” race group includes American Indian or Alaska Native, Native Hawaiian or other Pacific Islander, other, or multiple races. Comorbidities include chronic obstructive pulmonary disease (COPD), asthma, chronic respiratory disease, diabetes, congestive heart failure (CHF), advanced liver disease, or advanced renal disease. The GMTs for the subgroups are provided in Supplementary Tables 4 and 5.

The nAb responses to mRNA-1345 vaccination across prespecified subgroups, including frail and older participants and those with underlying comorbidities including CHF or COPD (considered at higher risk of RSV), were generally consistent with those of the overall PPIS for both RSV-A and RSV-B subtypes at day 29 (Figure 3, Supplementary Tables 4 and 5). The GMFRs (95% CIs) in participants aged 60–69 (9.8 [9.0–10.7]) years against RSV-A were higher than, and those for older adults 70–79 (7.5 [6.9–8.1]) years and ≥80 (8.1 [7.0–9.4]) years were comparable to, that of the overall PPIS (8.4 [8.0–8.9]). Across all subgroups, day 29 nAb GMFRs against the RSV-B subtype were consistent with that of the overall PPIS. The day 29 GMTs (95% CIs) against RSV-B were also generally consistent with those of the overall PPIS (7246.0 [6864.8–7648.4]) apart from lower GMTs (6164.0 [5715.6–6647.7]) in the non-Hispanic or Latino ethnicity group and higher GMTs (8476.4 [7865.3–9135.0]) for the Central/Latin America/Africa World Bank Region group. The SRRs across the age groups 60–69, 70–79, and ≥80 years were comparable to the overall cohort, as were the proportions of participants who achieved ≥2-fold titer increases from baseline versus the overall cohort for both RSV-A and RSV-B subtypes.

#### Binding Antibody

**Figure 4.**
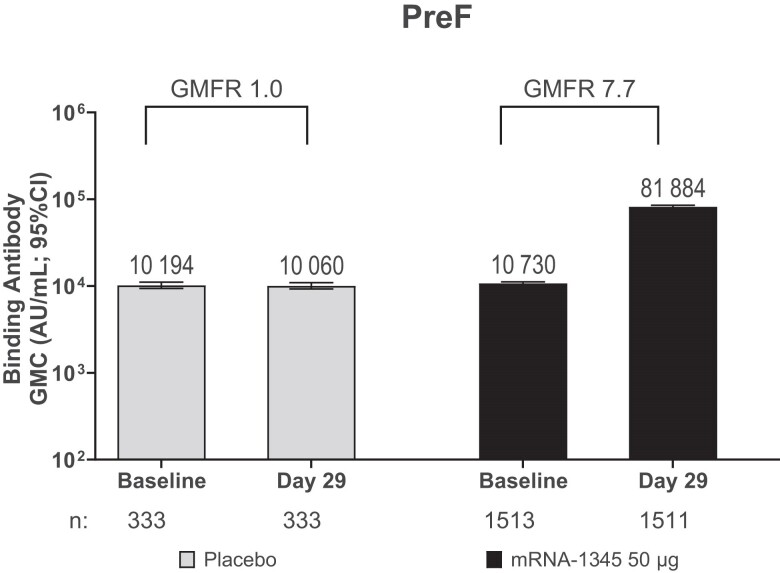
The geometric mean concentrations (GMCs; arbitrary units [AU]/mL) of binding antibodies to respiratory syncytial virus (RSV) prefusion F (preF) antigen were determined in a quantitative multiplex bead-based Luminex assay. Antibody values reported as below the lower limit of quantification (LLOQ; 35 for preF) are replaced by 0.5× LLOQ. Values greater than the upper limit of quantification (ULOQ; 580 553 for preF) are replaced by the ULOQ. The 95% confidence intervals (CIs) were calculated based on the *t*-distribution of the log-transformed values or the difference in the log-transformed values for GMC and geometric mean fold rise (GMFR), respectively, then back-transformed to the original scale for presentation. Brackets represent GMFRs from baseline determined for GMC at day 29 vs baseline; “n” indicates number of participants with nonmissing data at the visit (baseline or postbaseline).

Baseline preF bAb GMCs (95% CIs) were comparable between the mRNA-1345 (10 729.5 [10 310.6–11 165.5]) and placebo (10 194.3 [9374.5–11 085.7]) groups (Figure 4, Supplementary Table 6). At day 29, preF bAb GMCs in the mRNA-1345 group were higher (81 884.2 [78 644.2–85 257.6]) compared with placebo (10 060.2 [9258.9–10 930.7]), with a GMFR (95% CI) of 7.7 (7.3–8.0); the GMCs in the placebo group remained similar to baseline, with a GMFR of 1.0 (1.0–1.0). The preF bAb SRRs (95% CIs) at day 29 were 79.1% (77.0%–81.2%) and 0.3% (0.0%–1.7%) in the mRNA-1345 and placebo groups, respectively, and 94.2% (92.9%–95.4%) and 0.9% (0.2%–2.6%) of participants achieved a ≥2-fold increase in GMC from baseline.

**Figure 5.**
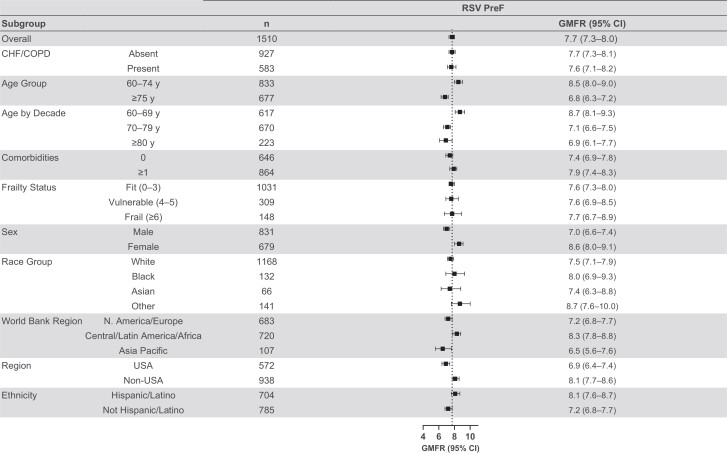
Prefusion F (PreF) binding antibody geometric mean concentrations (GMCs) were assessed in the per-protocol immunogenicity set (PPIS). GMCs (AU/mL) of binding antibodies for respiratory syncytial virus (RSV) preF in participants in the PPIS were determined in a quantitative multiplex bead-based Luminex assay. Geometric mean fold rises (GMFRs) were determined for GMCs at day 29 vs baseline. The 95% confidence intervals (CIs) were calculated based on the *t*-distribution of the log-transformed values or the difference in the log-transformed values for GMC and GMFR, respectively, then back-transformed to the original scale for presentation. Dotted line represents the overall GMFR for RSV preF; “n” indicates number of participants with nonmissing data at the visit (baseline or postbaseline). Age and risk factors were collected on electronic case report forms. Assignment to vaccination groups was stratified by age (60–74 y vs ≥75 y) and risk factors for lower respiratory tract disease (present vs absent). Baseline values for Edmonton Frail Scale total score were defined as the most recent nonmissing measurement (scheduled or unscheduled) collected on or before the date of injection of mRNA-1345 or placebo. “Other” race group includes American Indian or Alaska Native, Native Hawaiian or other Pacific Islander, other, or multiple races. Comorbidities include chronic obstructive pulmonary disease (COPD), asthma, chronic respiratory disease, diabetes, congestive heart failure (CHF), advanced liver disease, or advanced renal disease.

The preF bAb responses were also assessed among the same subgroups evaluated for nAbs. The preF bAb GMFRs and GMCs at day 29 for these subgroups were generally consistent with those of the overall PPIS, including those considered at higher risk of RSV (Figure 5, Supplementary Table 7). PreF bAb GMCs (95% CIs) were numerically higher in participants of Hispanic or Latino ethnicity (90 222.2 [85 323.5–95 402.0]), those with CHF/COPD (92 695.7 [86 844.8–98 940.7]), and those in the Central/Latin America/Africa World Bank Region (91 025.7 [86 061.7–96 276.0]) than the overall PPIS (81 884.2 [78 644.2–85 257.6]). The day 29 GMCs of participants with no baseline comorbidities of interest were numerically lower (73 099.9 [95% CI, 68 809.6–77 657.7]) than those of the overall PPIS.

## DISCUSSION

The day 29 immunogenicity results of the ConquerRSV trial demonstrate that vaccination with mRNA-1345 enhances levels of prevaccination nAbs against both RSV-A and RSV-B subtypes, and bAbs against the preF across demographic and high-risk subgroups compared with placebo. Day 15 immunogenicity results point to a rapid and high response following mRNA-1345. The mRNA-1345 enhancement of RSV-specific antibodies in older adults supports the clinical efficacy previously demonstrated against RSV disease in older adults [28].

The range of baseline nAb values observed in the trial reflects an adult population with prior exposure to RSV. Seroresponse was defined as a ≥4-fold increase in antibody levels from prevaccination levels, which is a standard in vaccine studies [33]. Prior exposure to antigen can lead to high baseline antibody levels among individuals that may influence responses to vaccination, and therefore, the ability to achieve SRRs as defined [33–35]. This also highlights that these individuals have higher circulating nAbs available at the time of vaccination. An analysis of SRRs showed that baseline GMTs against both RSV-A and RSV-B levels were lower among seroresponder participants who met these criteria than those who did not, indicating that levels of circulating preexisting nAbs were higher among nonseroresponders. Given the variability in antibody baseline levels, thresholds such as a ≥2-fold rise from baseline nAb levels have also been used in vaccine studies to avoid underestimation of the benefit of small increases in antibody titers [33]. In our study, the proportions of participants who achieved prespecified criteria of a ≥2-fold rise from baseline in antibody values were higher for both RSV-A (91.4%) and RSV-B (84.3%) than when assessed as a ≥4-fold rise in nAb values. Taken together, these data show that mRNA-1345 can enhance nAb levels among older adults with varying preexisting antibody levels, and that preexisting antibody levels can influence vaccination SRRs, consistent with prior studies [33–35].

Natural infection with RSV does not confer lifelong protection, implying that revaccination would be required after primary vaccination. The need and timing for revaccination with RSV vaccines is being evaluated in studies [36]. The mRNA-1345 vaccine, administered in a phase 1 study as a booster dose at 12 months, increased RSV-A and RSV-B nAb and preF bAb levels; titers after revaccination were numerically lower than those after the first dose, but with overlapping 95% CIs [29, 30], and mRNA-1345 revaccination is being further evaluated in this and other phase 3 studies (ClinicalTrials.gov: NCT04528719, NCT05127434, NCT05330975).

The RSV-A and RSV-B subtypes can co-circulate or be present separately as predominant subtypes during epidemic seasons and are differentiated on the basis of antigenic and genetic variability attributed mainly to the RSV attachment G protein [37, 38]. The overall conservation of amino acid sequences between the RSV-A and RSV-B types for the G protein is limited (53%) and much higher (≥90%) for the F glycoprotein [39, 40]. The preF conformation is the main humoral antigen target for RSV and displays all known neutralizing epitopes against both RSV-A and RSV-B [19, 20, 22, 41, 42]. The mRNA-1345 vaccine, encoding a stabilized preF protein derived from an RSV-A strain (RSV-A A2), enhanced nAb titers at day 29 against both RSV-A and RSV-B subtypes as expected, given the high conservation of the F protein. Increases in nAbs from baseline and SRRs were higher for RSV-A than those for RSV-B, consistent with numerically higher differences previously reported in the mRNA-1345 phase 1 study [30, 43]. The biological relevance of these findings is not fully understood and may be assay-related or due to differences in prior exposure to the subtypes, although it should be noted that the point estimate of mRNA-1345 vaccine efficacy against LRTD associated with RSV-A was higher than that associated with RSV-B in the phase 2/3 study; however, the mRNA-1345 vaccine efficacies against RSV-associated LRTD (≥2 and ≥3 signs or symptoms) for RSV-A and RSV-B were comparable to the overall cohort [28]. While this study did not evaluate T-cell responses, in a small study of adults 50–75 years, mRNA-1345 elicited strong and persistent T-cell CD4 and CD8 responses; evaluation of cell-mediated responses continues in additional studies [44]. Nonetheless, preF RSV vaccines, including mRNA-1345, reassuringly induce cross-neutralization and protection against both RSV subtypes [23, 26, 28, 30, 45]. The mRNA platform has the flexibility to evaluate other RSV formulations if such a need arises in the future.

mRNA-1345 also enhanced bAbs against the preF conformation at day 29 versus baseline levels, with overall response patterns generally similar to those shown for nAbs, including SRRs. The immune responses for nAb and preF bAb are consistent with the reported immunogenicity and efficacy of mRNA-1345 [28–30] and of RSV vaccines in development in clinical trials based on a preF strategy [21] that have demonstrated potent nAb responses and prevention of RSV [21, 23, 26, 28, 30, 45].

Consistent with the overall study cohort, mRNA-1345 enhanced nAb and bAb responses against RSV across demographic and risk groups. Immune responses remained generally comparable, with no clinically meaningful differences regardless of sex, race, and ethnicity, as well as among frail, older individuals, and those with underlying cardiovascular, endocrine, and respiratory conditions considered at higher risk of severe RSV-LRTD. RSV-A and RSV-B nAb GMTs in age groups (60–69, 70–79, and ≥80 years) were generally similar to those of the overall cohort, with overlapping 95% CIs. SRRs and proportions of participants achieving ≥2-fold increases from baseline were also comparable to the study cohort. These data indicate that mRNA-1345 provides protection against RSV in older persons who are predisposed to severe disease, attributed to age-related immunosenescence and underlying comorbidities [11]. Overall, the immunogenicity results support the earlier findings of broad protection against RSV disease across various populations, including those at severe risk of disease afforded by mRNA-1345 vaccination [28], consistent with prior studies, which have shown that induction of higher levels of nAb in high-risk adults can reduce disease severity and hospitalization during RSV infection [16].

A strength of this randomized study is the assessment of mRNA-1345 immunogenicity in a relatively large population across various demographic and risk groups for RSV. Limitations include the assessment of immunogenicity data at baseline and day 29 in this report; results from later time points being evaluated in the ongoing study may be reported at a future date. While both RSV-A and RSV-B were neutralized in the study, neutralization assays for the subtypes are distinct; therefore, direct comparison of absolute GMTs and GMFRs between RSV-A and RSV-B should be interpreted with caution. Additionally, immunocompromised individuals were not included, and immunogenicity was not evaluated separately in groups of participants with severe LRTD; however, the immune responses observed in subgroups of interest, including older age and frailty groups, as well as those with underlying conditions and comorbidities, are consistent with the efficacy previously demonstrated in these groups [28]. The number of participants with RSV-ARD eligible for inclusion in the PPIS was small, and this group was not evaluated separately; additional analysis of the relationship between immunogenicity and both LRTD and ARD groups is ongoing in the study. While the immunogenicity results consistently support the efficacy results, the relationship between immunogenicity and efficacy was not evaluated in this analysis and may be further assessed in the ongoing trial.

In conclusion, mRNA-1345 enhanced RSV-A and RSV-B nAb and preF bAb levels across subgroups, including those considered at higher risk of severe RSV disease. These immunogenicity data are consistent with the demonstrated efficacy of mRNA-1345 in the prevention of RSV across a spectrum of disease populations, which was reported in the primary analysis of the trial [28].

## Supplementary Data

Supplementary materials are available at *The Journal of Infectious Diseases* online (http://jid.oxfordjournals.org/). Supplementary materials consist of data provided by the author that are published to benefit the reader. The posted materials are not copyedited. The contents of all supplementary data are the sole responsibility of the authors. Questions or messages regarding errors should be addressed to the author.

## Supplementary Material

jiae316_Supplementary_Data

## Notes

***Acknowledgments.*** We thank the participants in the trial and the members of the mRNA-1345 trial team (listed in the Supplementary Materials) for their dedication and contributions to the trial and Frank J. Dutko (Moderna consultant) for development of data displays and editorial support.

***Author contributions.*** All authors read and approved the final manuscript.

***Data availability.*** As the trial is ongoing, access to patient-level data and supporting clinical documents with qualified external researchers will be made available upon request and subject to review the trial completion date. A materials transfer and/or data access agreement with the sponsor will be required for accessing shared data. Such requests can be made to the corresponding author.

***Financial support*.** This study was funded by Moderna, Inc.
