# Supplementary material for: Humoral Immunogenicity of mRNA-1345 RSV Vaccine in Older Adults
Source: J Infect Dis. 2024 Jun 18;230(5):e996–e1006. doi: 10.1093/infdis/jiae316 (PMC11566230; doi:10.1093/infdis/jiae316)
Supplement: jiae316_Supplementary_Data [file jiae316_supplementary_data.docx]

# **Supplementary Appendix**

# **List of ConquerRSV Investigators**

| **Name** | **Institute** | **Location** |
| --- | --- | --- |
| Aazami, Hessam | Hope Clinical Research, LLC | Kissimmee, FL, USA |
| Abbott, Joanna | Synexus Hexham Clinical  Research Centre | Corbridge, Hexam, UK |
| Abitbol, Alexander | Centricity Research | Toronto, Canada |
| Acevedo Diaz, Elia Enid | CBH Health - CenExel CBH | Gathersburg, MD, USA |
| Agarwal, Puneet | Synexus Scotland Clinical  Research Centre | Bellshill, Glasgow, Scotland |
| Ahmed, Salahuddin | Johns Hopkins University | Zakiganj and Sylhet,  Bangladesh |
| Allaw, Mohammed | Synexus Clinical Research US,  Inc. | Evansville, IN, USA |
| Alvarado, Hilario | Synexus Clinical Research US,  Inc. | San Antonio, TX, USA |
| Ampajwala, Madhavi | Village Health Partners Frisco  Medical Village | Frisco, TX, USA |
| Anderson, Duane | Excel Clinical Research | Las Vegas, NV, USA |
| Badenhorst, Josias Servaas | CARe Clinic | Red Deer, AB, Canada |
| Barnett, Leanne | New Zealand Clinical Research | Auckland, New Zealand |
| Baron, Mira | Palm Beach Research | West Palm Beach, FL, USA |
| Bautista Toloza, Leonardo | Centro de Atención e  Investigación Médica S.A. | Bogotá, Cundinamarca,  Colombia |
| Bergthold, James | Summit Research Network,  Inc. | Portland, OR, USA |
| Berman, Gary | Clinical Research Institute, Inc | Minneapolis, MN, USA |
| Bernstein, Richard | Privia Medical Group, LLC | Annapolis, MD, USA |
| Berteau, Tammy | Diex Recherche | Québec City, QC, Canada |
| Bickel, Markus | Infektioresearch GmbH & Co.  KG | Frankfurt am Main, Germany |
| Bisshop, Fiona | Holdsworth House Medical | QLD, Australia |
| Blank, Stephen | Mount Vernon Clinical  Research, LLC | Atlanta, GA, USA |
| Boone, Gary | Paradigm Clinical Research  Institute Inc | Redding, CA, USA |
| Bordon, Jose | Washington Health Institute | Washington, DC, USA |
| Borobia Perez, Alberto | Hospital Universitario La Paz | Madrid, Spain |
| Bradley, Paul | Velocity Clinical Research | Savannah, GA, USA |
| Brahmbhatt, Gaurang | Riverside Medical Group -  Circuit | Hoboken, NJ, USA |
| Brazg, Ronald | Rainier Clinical Research  Center | Renton, WA, USA |
| Breedt, Johannes | Dr. J Breedt | City of Tshwane, Gauteng,  South Africa |
| Brimberry, Matthew | ACRC Trials | Plano, TX, USA |
| Brosz, Adam | Velocity Clinical Research | Grand Island, NE, USA |
| Brown, Mary | DM Clinical Research | River Forest, IL, USA |
| Buntinx, Erik | ANIMA Research Center | Diapenbeek, Limburg, Belgium |
| Burgher, Abram | Hope Research Institute LLC | Hunt, AZ, USA |
| Butcher, Michael | Velocity Clinical Research | Cincinnati, OH, USA |
| Buynak, Robert | Velocity Clinical Research | Valparaiso, IN, USA |
| Campos Barker, Maria | Biomedica Research Group | Valparaíso, Chile |
| Carrillo Castro, Jaime | Centro de Atención e  Investigación Médica S.A. | Cundinamarca, Colombia |
| Casey, Sherri | Benchmark Research | Covington, LA, USA |
| Cesarz, Marek | Synexus Polska Sp. z o.o.  Oddzial w Gdansku | Pomorskie, Poland |
| Chan, Yvonne Fu Zi | Singapore General Hospital | Singapore, Singapore |
| Chandler, Reynaldo | CEVAXIN 24 de diciembre | Panamá, Panama |
| Chang, Margaret | Velocity Clinical Research | Edgewater, FL, USA |
| Chaparro, Gustavo | Instituto Medico Platense | Buenos Aires, Argentina |
| Cheng, Huey-Shinn | Chang Gung Memorial Hospital | Taoyuan City, Taiwan |
| Choi, Jun Yong | Severance Hospital Yonsei  University Health System | Seoul, Republic of Korea |
| Choi, Min Joo | Catholic Kwandong University  International St. Mar | Incheon, Republic of Korea |
| Choi, Melissa | Synexus Clinical Research US,  Inc. | Richfield, MN, USA |
| Choo, Eun-Ju | Soon Chun Hyang University  Hospital | Bucheon-si, Republic of Korea |
| Clark, Rebecca | Layton Medical Centre | Blackpool, Lancashire, UK |
| Cohen, Harris | Hatboro Medical Associates | Hatboro, PA, USA |
| Colville, Bonnie | DM Clinical Research - Texas  Center For Drug Development | Houson, TX, USA |
| Conradie, Sonja | Synexus Watermeyer Clinical  Research Centre | Pretoria, Gauteng, South Africa |
| Contzen, Christel | Synexus Frankfurt Research  Centre | Hessen, Germany |
| Cotugno, Michael | Benchmark Research | Metairie, LA, USA |
| Cupelo, Robert | Velocity Clinical Research | East Syracuse, NY, USA |
| Damian Hernandez, Matilde | CEMDEC | Mexico City, Guadalajara,  Mexico |
| Davis, Matthew | Rochester Clinical Research,  Inc | Rochester, NY, USA |
| Davis, Joseph | Hope Research Institute LLC | Hunt, AZ, USA |
| De Francisco, Don | PRI, LLC | Newport Beach, CA, USA |
| De Valle, Oscar | West Houston Clinical  Research | Houston, TX, USA |
| Denham, Douglas | Flourish Research | San Antonio, TX, USA |
| Dennis, Patrick | DelRicht Clinical Research, LLC | New Orleans, LA, USA |
| Desai, Nitinchandra | Carolina Institute for Clinical  Research | Fayettevill, NC, USA |
| Deseda, Carmen | Caribbean Medical Research  Center | San Juan, Puerto Rico |
| Doehner, Tamara | CCT Research | Fremont, NE, USA |
| Doreski, Pablo Alexis | Fundación Respirar - Vaccine  Research Division | Buenos Aires, Argentina |
| Doust, Matthew | Hope Research Institute LLC | Phoenix, AZ, USA |
| Elizondo Moreno, Eduardo | Accelerium, S. de R.L. de C.V. | Durango, Mexico |
| Elzi, Stanton | Paradigm Clinical Research  Institute Inc | Redding, CA, USA |
| Ensz, David | Velocity Clinical Research | Sioux City, IA, USA |
| Fausett, Merlin | Boeson Research MSO | Missoula, MT, USA |
| Fierro, Carlos | Johnson County Clin-Trials | Lenexa, KS, USA |
| Finley, Jon | Clinical Research Atlanta | Stockbridge, GA, USA |
| Fitzgibbons, William | Skyline Medical Center, PC | Elkhorn, NE, USA |
| Fitz-Patrick, David | East West Medical Research  Institute | Honolulu, HI, USA |
| Flanagan, Clinton | Tekton Research | Longmont, CO, USA |
| Focil, Augusto | FOMAT Medical Research | Oxnard, CA, USA |
| Freeman, George | Health Research of Hampton  Roads Inc. | Newport News, VA, USA |
| Froer, Michael | Klinische Forschung, Schwerin  GmbH (KFGN) | Schwerin, Mecklenburg-  Vorpommern, Germany |
| Fujino, Minoru | Boocs Clinic Fukuoka | Fukuoka, Japan |
| Fuller, Gregory | Helios Clinical Research, Inc. | New York, NY, USA |
| Fuller, Ashley | Seattle Clinical Research  Center | Seattle, WA, USA |
| Garcia Gonzalez, Maria del  Carmen | Instituto de Investigación en  Ciencias Médicas S.A. | San José, Puerto Rico |
| Garcia Ortiz, Luis Hernando | IPS Centro de Investigacion  Clinicas Cardiomet | Risaralda, Colombia |
| Gerdis, Michael | Drug Trials America | Hartsdale, NY, USA |
| Girard, Ginette | Diex Recherche | Sherbrooke, Québec, Canada |
| Glaves, Matthew | Synexus Wales Clinical  Research Centre | Cardiff, UK |
| Goecke Hochberger, Bernardo | Hospital Base de Osorno | Osorno, Los Lagos, Chile |
| Götze, Marie-Annick | VAXINFECTIO University of  Antwerp - Center for the Evaluation of Vaccination | Antwerp, Belgium |
| Grayson-Mathis, Charlotte | Javara, Inc./Privia Medical  Group Georgia, LLC | Fayetteville, GA, United States |
| Guillory, Clinton | IMA Clinical Research | Monroe, LA, USA |
| Gunchev, Desislav | Synexus Midlands Clinical  Research Centre | Birmingham, West Midlands,  UK |
| Guzman Romero, Ana Karla | RM Pharma Specialists | Chihuahua City, Mexico |
| Han, Sang Hoon | Gangnam Severance Hospital,  Yonsei University Health System | Seoul, Republic of Korea |
| Haranaka, Miwa | SOUSEIKAI PS Clinic | Fukuoka, Japan |
| Harvey, James | Privia Medical Group | Fort Worth, TX, USA |
| Hazlewood, Christopher | Southern Clinical Trials Limited | Christchurch, New Zealand |
| Hemmersmeier, John | South Ogden Family Medicine | South Ogden, UT, USA |
| Henderson, Jeffrey | American Indian Clinical Trials  Research Network | Rapid City, SD, USA |
| Herrera, Gisela | Hospital CIMA | San José, Puerto Rico |
| Heurich, Eva-Maria | Accel Research Sites | Maitland, FL, USA |
| Higuera, Juan | Fundacion Oftalmologica de  Santander Foscal | Floridablanca, Colombia |
| Holloway, Lisa | DM Clinical Research | Sugarland, TX, USA |
| Holloway, Lisa | DM Clinical Research | Sugarland, TX, USA |
|  |  |  |
| Hussen, Nazreen Jeewa | Worthwhile Clinical Trials | Benoni, South Africa |
| Iglesias Pertuz, Shirley Patricia | Clinica de la Costa Ltda | Atlántico, Colombia |
| Irfan, Muhammad | DM Clinical Research | Tomball, TX, USA |
| Janik, Konrad | Synexus Berlin Research  Centre | Berlin, Germany |
| Jethani, Manohar | Affinity Health | Oak Brook, IL, USA |
| Jimenez, Gilberto | Spotlight Research Center, LLC | Miami, FL, USA |
| Johnson, Kimball | iResearch | Decatur, GA, USA |
| Jung, Dong Sik | Dong-A University Hospital | Busan, Republic of Korea |
| Kasarjian, Julie | Velocity Clinical Research | Banning, CA, USA |
| Khetan, Shishir | Velocity Clinical Research | Rockville, MD, USA |
| Kim, Young Keun | Yonsei University Wonju  Severance Christian Hospital | Seoul, Republic of Korea |
| Kim, Yeon Sook | Chungnam National University  Hospital | Daejeon, Republic of Korea |
| Kim, Chung-Jong | Ewha Womans University  Seoul Hospital | Seoul, Republic of Korea |
| Ko, Jae-Hoon | Samsung Medical Center | Seoul, Republic of Korea |
| Kokko, Satu | FVR/Oulun  Rokotetutkimusklinikka | Oulu, Pohjois-Pohjanmaa,  Finland |
| Kondo, Hiroaki | Higashi Shinjuku Clinic | Shinjuku-Ku, Japan |
| Konieczny, Grzegorz | Synexus Polska Sp. z o.o.  Oddzial w Poznaniu | Poznan, Wielkopolskie, Poland |
| Koren, Michael | Jacksonville Center For Clinical  Research | Jacksonville, FL, USA |
| Koski, Susanna | FVR/Etelä Helsingin  Rokotetutkimusklinikka | Helsinki, Uusimaa, Finland |
| Laajalahti, Outi | FVR/Seinäjoen  Rokotetutkimusklinikka | Seinäjoki, Etelä-Pohjanmaa,  Finland |
| Leah, Amber | Paratus Clinical Research | Bruce, Australian Capital  Territory, Australia |
| Lee, Jin-Soo | Inha University Hospital | Incheon, Republic of Korea |
| Lee, Jacob | Hallym University Kangnam  Sacred Heart Hospital | Seoul, Republic of Korea |
| Letica, Arna | Optimal Clinical Trials Ltd | Auckland, New Zealand |
| Lin, Yi-Tsung | Taipei Veterans General  Hospital | Beitou District, Taipei City,  Taiwan |
| Liu, Chiu-Shong | China Medical University  Hospital | Taichung City, Taiwan |
| Liu, Po-Yu | Taichung Veterans General  Hospital | Taichung City, Taiwan |
| Lockwood, Robert | Tekton Research Inc | Yukon, OK, USA |
| Lopez, Eduardo | Fundación Socolinsky Centro  de Vacunación Proteger | Buenos Aires, Argentina |
| Lupercio, Rafael | Paradigm Clinical Research  Institute Inc | Redding, CA, USA |
| Malherbe, Elmien | Synexus Watermeyer Clinical  Research Centre | Gauteng, South Africa |
| Mansfield, Justin | Alpine Research Organization,  Inc | Clinton, UT, USA |
| Marcum, Benjamin | Privia Medical Group | Stephenville, TX, USA |
|  |  |  |
| Martinot, Jean-Benoît | Private Practice Dr Jean Benoit  Martinot | Erpent, Belgium |
| Maytham, Colleen | Okanagan Clinical Trials | Kelowna, British Columbia,  Canada |
| McGirr, Anthony | Northern Beaches Clinical  Research | New South Wales, Australia |
| Mehta, Saumil | AIM Trials | Plano, TX, USA |
| Mesotten, Dieter | Ziekenhuis Oost-Limburg | Limburg, Belgium |
| Meyer, Jay | Velocity Clinical Research | Lincoln, NE, USA |
| Middleditch, Alexander | Bristol Royal Infirmary | Avon, UK |
| Middleton, Randle | Optimal Research Alabama | Huntsville, AL, USA |
| Miller, Vicki | DM Clinical Research | Houston, TX, USA |
| Mohan, Rahul | Paratus Clinical Research | Sydney, New South Wales,  Australia |
| Molero Bonilla, Marta | Hospital Son Llatzer | Palma, Spain |
| Monroy Avella, Jorge | Flourish Research | Winter Park, FL, USA |
| Montero Arias, María Felicia | Hospital Clínica Bíblica Sede  Santa Ana | San José, Puerto Rico |
| Morfin, Maria del Rayo | Maria del Rayo Morfin Otero | Jalisco, Mexico |
| Murdoch, Louise | Emeritus Research | Victoria, Australia |
| Murray, Linda | Synexus Clinical Research US,  Inc. | Pinellas Park, FL, USA |
| Mutterperl, Robert | Tucson Neuroscience Research | Tucson, AZ, USA |
| Myneni, Banu | Velocity Clinical Research -  Family Practice | Portsmouth, VA, USA |
| Nakamura, Kenjiro | Tenjin Sogo Clinic | Fukuoka, Japan |
| Narejos Perez, Silvia | ABS Centelles | Barcelona, Spain |
| Nevarez Ruiz, Luis Alejandro | Investigacion en Salud y  Metabolismo S.C. | Chiapas, Mexico |
| Nixon, William | Lakeview Clinical Research | Guntersville, AL, USA |
| Nugent, Paul | Synexus Clinical Research US,  Inc | Cincinnati, OH, USA |
| Nunez, Margarita | Synexus Clinical Research US,  Inc. | New York, NY, USA |
| Ocampo Soto, Rebeca | Consultorio Medico La Flor | Guanacaste, Puerto Rico |
| Ochoa Castañeda, Paula | Centro de Atención e  Investigación Médica S.A. - CAIMED | Ibagué, Colombia |
| Ochoa Mazarro, Dolores | Hospital Universitario de La  Princesa | Madrid, Spain |
| Odekirk, Larry | Lynn Institute of Denver | Aurora, CO, USA |
| Okolie, Angela | Synexus Clinical Research US,  Inc. | Orlando, FL, USA |
| Olech-Cudzik, Anna | Ostrowieckie Centrum  Medyczne Spólka Cywilna Anna Olech-Cudzik, Krzysztof Cudzik | Swietokrzyskie, Poland |
| Oñate, José | Centro Medico Imbanaco de  Cali S.A. | Santiago De Cali, Colombia |
| Overcash, Jeffrey | Velocity Clinical Research | La Mesa, CA, USA |
| Ozunlu, Pembe | NW Consortium Merseyside | Lancashire, UK |
| Paavola, Pauliina | FVR/Kokkolan  Rokotetutkimusklinikka | Keski-Pohjanmaa, Finland |
| Packham, Jonathan | Cheadle Hospital | Staffordshire, UK |
| Padayachee, Trevenesan | Aliwal Shoal Medical Center | Kwazulu - Natal, South Africa |
| Paquette, Jean-Sebastien | Diex Recherche | Joilette, QC, Canada |
| Patel, Suchet | Velocity Clinical Research | Vestal, NY, USA |
| Patel, Nathan | DM Clinical Research | Southfield, MI, USA |
| Pavie Gallegos, Juana | Centro Respiratorio Integral  LTDA | Los Lagos, Chile |
| Pearce, Katherine | Velocity Clinical Research | Baton Rouge, LA, USA |
| Pearlman, Laura | Synexus Clinical Research US,  Inc. | Chicago, IL, USA |
| Pellegrino, Richard | Baptist Health Center for  Clinical Research | Little Rock, AR, USA |
| Pérez Breva, Lina | FISABIO-Public Health | Valencia, Spain |
| Perez Marc, Gonzalo | Hospital Militar Central  Cirujano Mayor Dr. Cosme Argerich | Buenos Aires, Argentina |
| Peterson, Bryce | Cope Family Medicine | Bountiful, UT, USA |
| Pickrell, Paul | Tekton Research Inc | Austin, TX, USA |
| Powell, Richard | Velocity Clinical Research | Cincinnati, OH, USA |
| Prada, Gonzalo | Solano & Terront Servicios  Médicos LTDA (UNIENDO) | Bogotá, Colombia |
| Pragalos, Antoinette | CTI Clinical Research Center | Norwood, OH, USA |
| Pretswell, Carol | NW Consortium Lancashire | Lancashire, UK |
| Price, David | Royal Victoria Infirmary | Northumberland, UK |
| Probst, Jared | Olympus Family Medicine | Holladay, UT, USA |
| Raiser, Frederick | Velocity Clinical Research | Omaha, NE, USA |
| Rämet, Mika | Finnish Vaccine Research | Tampere, Finland |
| Reddy, Mallu | Reddy Care Medical | Pomona, CA, USA |
| Reyes Fentanes, Maria Jose | PanAmerican Clinical Research | Querétaro, Mexico |
| Reynales, Humberto | Centro de Atención e Investigación Médica S.A. | Cundinamarca, Colombia |
| Rickner, Kyle | Tekton Research | Yukon, OK, USA |
| Rodriguez, Jesus | BRCR Global Puerto Rico | Ponce, Puerto Rico |
| Rodriguez Arenales, Edith | Centro de Atención e  Investigación Médica S.A. | Distrito Capital de Bogotá,  Colombia |
| Rosenberg, David | PRI, LLC | Los Alamitos, CA, USA |
| Roux, Susanna Magrieta | Synexus - Helderberg Clinical  Research Centre | Western Cape, South Africa |
| Saavedra Pacheco, Carla | BIOCINETIC SpA | Maule, Chile |
| Saez-Llorens, Xavier | CEVAXIN Avenida México | Panamá, Panama |
| Santana-Bagur, Jorge | Puerto Rico AIDS Clinical Trials  Unit | San Juan, Puerto Rico |
| Santiago, Fredric | DM Clinical Research - Texas  Center for Drug Development | Humble, TX, USA |
| Schaefer, Axel | Medizentrum Essen Borbeck -  HAND | Nordrhein-Westfalen,  Germany |
| Sekino, Hisakuni | Sekino Hospital | Toshima-Ku, Japan |
| Sellman, Richard | Montana Medical Research | Missoula, MT, USA |
| Seppä, Ilkka | FVR/Porin  Rokotetutkimusklinikka | Satakunta, Finland |
|  | FVR/Turun  Rokotetutkimusklinikka | Varsinais-Suomi, Finland |
|  | FVR/Tampereen  Rokotetutkimusklinikka | Pirkanmaa, Finland |
| Shaw, Marian | Velocity Clinical Research | Meridian, ID, USA |
| Shockey, Gerald | Desert Clinical Research, LLC | Mesa, AZ, USA |
| Sia, Ying Tung | Diex Recherche | Trois Rivieres, Québec, Canada |
| Silva, Rafael | Centro de Investigacion del  Maule | Talca, Chile |
| Simmons, Todd | Medical Center For Clinical  Research | San Diego, CA, USA |
| Singh, Raj | HEALOR Primary Care  Physicians | Las Vegas, NV, USA |
| Sirnelä-Rif, Elina | FVR/Itä Helsingin  Rokotetutkimusklinikka | Uusimaa, Finland |
| Smith, Susan | Waitemata Clinical Research | Auckland, New Zealand, |
| Smith, Brian | Versailles Family Medicine | Versailles, KY, USA |
| Soto, Efrain | Allure Health LLC | Friendswood, TX, USA |
| Sprague, Amy | Masters of Clinical Research  Inc | Augusta, GA, USA |
| Stephens, Michael | Fleming Island Center For  Clinical Research | Fleming Island, FL, USA |
| Stewart, Jeffrey | ACRC Trials | Plano, TX, USA |
| Strauss, Guy | Multi-Specialty Research  Associates, Inc. | Lake City, FL, USA |
| Surber, Joseph | Centricity Research | Roswell, GA, USA |
| Surowitz, Ronald | Health Awareness | Jupiter, FL, USA |
| Szymkowiak, Katarzyna | Synexus Polska Sp. z o.o.  Oddzial we Wroclawiu | Dolnoslaskie, Poland |
| Takatsuka, Yoji | Institute Medical Corporation  Hitomikai Motomachi Takatsuka Naika Clinic | Yokohama, Japan |
| Takeuchi, Yuzuru | Fukui General Hospital | Fukui-Shi, Japan |
| Tamer, Yousef | Synexus Scotland Clinical  Research Centre | Lanarkshire, UK |
| Tark, Marvin | Drug Studies America | Marietta, GA, USA |
| Thakor, Michael | Tekton Research | Fort Collins, CO, USA |
| Tharenos, Leslie | Synexus Clinical Research US,  Inc. | Creve Coeur, MO, USA |
| Thurlow, Claire | Southern Clinical Trials | Nelson, New Zealand |
| Tinoco Favila, Juan | Hospital General 450 | Durango, Mexico |
| Toma, Ramy | Synexus Clinical Research US,  Inc. | Birmingham, AL, USA |
| Trevor, Lucy | NW Consortium | Manchester, UK |
| Uchiyama, Akiyoshi | Medical Corporation Asbo  Tokyo Asbo Clinic | Tokyo, Japan |
| Ujiie, Mugen | Center Hospital of the National  Center for Global Health and Medicine | Shinjuku-Ku, Japan |
| Ukkonen, Benita | FVR/Espoon  Rokotetutkimusklinikka | Uusimaa, Finland |
| Uribe, Eduardo | PanAmerican Clinical Research  LLC | Brownsville, TX, USA |
| Vale, Noah | Centricity Research | Toronto, Canada |
| Vallieres, Gerald | Centricity Research | Québec, Canada |
| Varano, Susann | Clinical Research Consultants  LLC | Milford, CT, USA |
| Vidiella, Gabriela | Swiss Medical Center Barrio  Parque | Buenos Aires, Argentina |
| Vijil, Julio | Velocity Clinical Research | Hallandale Beach, FL, USA |
| Viljoen, Marianne | NW Consortium | Manchester, UK |
| Virta, Miia | FVR/Järvenpään  Rokotetutkimusklinikka | Uusimaa, Finland |
| Voloshyna, Olga | Northside Health | New South Wales, Australia |
| Watanabe, Go | NewHeart Watanabe Institute | Suginami City, Japan |
| Waters, Michael | Velocity Clinical Research | Chula Vista, CA, USA |
|  | IMA Clinical Research | St. Petersburg, FL, USA |
|  | Clinical Trials of Tampa | Tampa, FL, USA |
|  | Affinity Health | Nashville, TN, USA |
| Weinstein, Debra | IMA Clinical Research | Manhattan, NY, USA |
|  | Triad Clinical Trials | Greensboro, NC, USA |
|  | Randomize Now | Peachtree City, GA, USA |
|  | IMA Clinical Research | Raritan, NJ, USA |
|  | PPD Virtual - Science 37, Inc | Culver City, CA, USA |
| Wijewardane, Priyantha | Baptist Health Center for  Clinical Research | Little Rock, AR, USA |
| Williams, Michael | Lakeland Clinical Trials | Rotorua, New Zealand |
| Williams, Barton | Trial Management Associates  LLC | Wilmington, NC, USA |
| Williams, Hayes | Accel Research Site | Birmingham, AL, USA |
| Williamson, Derrick | Great Lakes Research Institute | Southfield, MI, USA |
| Wolf, Thomas | Methodist Physicians Clinic | Fremont, NE, USA |
| Yenal, Kem | DM Clinical Research | Philadelphia, PA, USA |
| Yousef, Tamer | Synexus Scotland Clinical  Research Centre | Lanarkshire, UK |
| Zacher, Jeffrey | Synexus Clinical Research US,  Inc | Phoenix, AZ, USA |
| Zaczek-Chmielewska, Monika | Synexus Polska Sp. z o.o.  Oddzial w Warszawie | Gdańsk, Poland |
| Zakrzewski, Marcin | Synexus Polska Sp. z o.o.  Oddzial w Katowicach | Slaskie, Poland |
| Zaman, Khalequz | Icddr,b Kamalapur | Dhaka, Bangladesh |
|  | icddr,b Matlab Health  Research Center | Matlab Bazar, Bangladesh |
| Zizzo, Steven | Dr. Steven Z. Zizzo Medicine Professional Corporation | Toronto , Canada |

# **Supplemental Methods**

## ***Inclusion and Exclusion criteria***

*Inclusion Criteria*

Participants eligible to be included in the study were:

1. Adults ≥60 years of age who were primarily responsible for self-care and activities of daily living. Participants could have had one or more chronic medical diagnoses (including CHF [including heart failure with preserved ejection fraction] and COPD), but should be medically stable as assessed by the following criteria:

− Absence of changes in medical therapy within 1 month due to treatment failure or toxicity

− Absence of medical events qualifying as SAEs within 1 month of the planned study injection on day 1

− Absence of known, current, and life-limiting diagnoses, which could continue for the duration of the primary efficacy period (12 months from study injection on day 1) and which, in the opinion of the investigator, would make completion of the protocol unlikely.

2. Body mass index from ≥18 kg/m2 to ≤35 kg/m2.

3. Willing and able (on both physical and cognitive basis) to give informed consent prior to study enrollment.

4. Able to comply with study requirements.

*Exclusion Criteria*

Participants were excluded from the study if any of the following criteria applied:

1. Participation in another clinical research study where participant has received an investigational product (drug/biologic/device with the exception of investigational RSV products) within 6 months before the planned date of the day 1 study injection. Current participation in another RSV investigational study was exclusionary.

2. History of a diagnosis or condition that, in the judgment of the investigator, was clinically unstable or could affect participant safety, assessment of safety endpoints, assessment of immune response, or adherence to study procedures. Clinically unstable was defined as a diagnosis or condition requiring significant changes in management or medication within the 2 months prior to screening and included ongoing workup of an undiagnosed illness that could lead to a new diagnosis or condition.

3. Reported history of congenital or acquired immunodeficiency, immunosuppressive condition, or immune-mediated disease. Note: Human immunodeficiency virus (HIV) positive participants with CD4 count ≥ 350 cells/mm3 and an undetectable HIV viral load within the past year (low level variations from 50-500 viral copies which do not lead to changes in antiretroviral therapy) as determined from participant’s medical records, are permitted. To clarify, participants with stable autoimmune diseases that do not require systemic immunosuppressants (per Exclusion Criterion #9) are permitted.

4. Dermatologic conditions that could affect local solicited AR assessments (eg, tattoos, psoriasis patches affecting skin over the deltoid areas).

5. Reported history of anaphylaxis or severe hypersensitivity reaction after receipt of the mRNA-1345 vaccine or any components of the mRNA-1345 vaccine.

6. Reported history of bleeding disorder that is considered a contraindication to IM injection or phlebotomy.

7. History of a serious reaction to any prior vaccination, or Guillain-Barré syndrome within 6 weeks of any prior influenza immunization.

8. Received or planned to receive any nonstudy vaccine (including authorized or approved vaccines for the prevention of COVID-19 regardless of type of vaccine) within 28 days before or after the day 1 study injection. Nonstudy vaccination(s) should not be delayed.

9. Chronic administration (defined as more than 14 continuous days) of immunosuppressants or other immune-modifying drugs within 6 months prior to the administration of the study injection. An immunosuppressant dose of glucocorticoid was defined as a systemic dose ≥10 mg of prednisone per day or equivalent. The use of topical, inhaled, and nasal glucocorticoids was permitted.

10. Administration of immunoglobulins and/or any blood products within the 3 months preceding the administration of the study injection or during the study.

11. Acute disease at the time of enrollment (defined as the presence of moderate or severe illness with or without fever, or an oral temperature ≥37.8°C (100.0°F) on the planned day of vaccine administration).

12. Any medical, psychiatric, or occupational condition, including reported history of drug or alcohol abuse, that, in the opinion of the investigator, might pose additional risk due to participation in the study or could interfere with the interpretation of study results.

13. Known history of poorly controlled hypertension (per determination of the investigator), or systolic blood pressure >160 mmHg at the Screening or baseline (day 1) visit.

14. Known history of hypotension, or systolic blood pressure <85 mmHg at the screening or baseline (day 1) visit.

15. Diastolic blood pressure >90 mmHg at the Screening or baseline (day 1) visit. Known uncontrolled disorder of coagulation.Note: Participants receiving aspirin, clopidogrel, prasugrel, dipyridamole, dabigatran, apixaban, rivaroxaban, or warfarin for cardiovascular prophylaxis or prophylaxis of thromboembolic disease or stroke in the setting of atrial fibrillation and under good control were NOT excluded.

17. History of myocarditis, pericarditis, or myopericarditis within 2 months prior to Screening. Participants who have not returned to baseline after their convalescent period were also excluded.

18. Donated ≥450 mL of blood products <14 days prior to screening.

19. Member of study personnel or an immediate family member or household member of

study personnel.

Participants with comorbidities of interest in addition to COPD and CHF who were medically stable including asthma, any chronic respiratory or pulmonary disease, diabetes mellitus type 1 or 2, advanced liver and renal diseases were also included. Participant frailty was assessed using the Edmonton Frail Scale[1] across 9 domains: cognition, general health status, functional independence, social support, medication use, nutrition, mood, continence, and functional performance. The participant’s frailty status will be assessed at baseline, 12 months, and 24 months, and recorded on the appropriate eCRF page.

## ***Sampling and Selection of Random Immunogenicity Subcohort***

Immunogenicity was assessed in the Per-protocol Immunogenicity (PPI) Set, a randomly selected subset of participants in the Random Immunogenicity Subcohort who were randomized and dosed by 31 Oct 2022 in the trial. Using this subcohort ensured that the PPI Set was aligned with the population contributing to the primary analysis of efficacy (30 Nov 22 data cutoff date). The PPI Set consisted of participants in the Random Immunogenicity Subcohort who received the assigned study vaccine dose according to protocol and had RSV immunogenicity titer results at baseline (prior to study vaccine administration) with at least 1 valid result after the study vaccine administration at the timepoint of interest and had no major protocol deviations affecting the primary immunogenicity outcomes as determined prior to database lock and unblinding. The PPI Set criteria did not exclude participants identified with an event of RSV-ARD or RSV‑LRTD post-baseline. The Random Immunogenicity Subcohort selected participants in a 5:1 ratio of mRNA-1345 to placebo. The stratified selection of participants in the Random Immunogenicity Subcohort ensured adequate representation by age (60 to 74 years and ≥75 years), LRTD risk factor (absent/present), and region (Northern/Southern hemisphere) (Table S1).

The sampling process was based on the same data cut (30-APR-2023) used for the first interim analysis (IA1) reported in the primary efficacy analysis.[2] A total of ~35538 participants (~96% of the planned sample size of 37000), which was adequately representative of the trial population, was included for sampling. This approach preserved the consistency of participant population between the IA1 and the immunogenicity analyses, and also provided the necessary time to process the immunogenicity samples without delaying subsequent trial activities as the sampling process could start before the trial reached the full enrollment. The Random Immunogenicity Subcohort was selected using a stratified random sampling design. Random sampling was stratified by key baseline characteristics (assigned treatment arm, stratification factors used for randomization, and region of Northern and Southern Hemisphere). This sampling design enabled characterization of immune response (bAb and nAb readouts) in all relevant subgroups.

The table below describes the planned number of participants to be randomly sampled from each of 7 strata defined based on the randomization strata and geographic region. A total of ~2,128 participants was planned, based on adjusting for approximately 10% of participants who may be excluded from the Per-protocol Immunogenicity Set, may not have results due to issues with serum samples or assay processing, etc. with a planned target of approximately 1,920 participants in the per-protocol immunogenicity subset.

| **Numbers of Participants Randomly Sampled into the Random Subcohort** | | | | | | | |
| --- | --- | --- | --- | --- | --- | --- | --- |
|  | Number of Participants in Per-protocol Immunogenicity Set (Total N=1920) | | | | | | |
| Baseline Covariate Strata^a^ | S1 | S2 | S3 | S4 | S5 | S6 | S7 |
| mRNA-1345 50 µg | 200 | 200 | 400 | 200 | 200 | 200 | 200 |
| Placebo | 40 | 40 | 80 | 40 | 40 | 40 | 40 |
|  | Number of participants in Random Subcohort (Total N=2128) | | | | | | |
| mRNA-1345 50 µg | 222 | 222 | 444 | 222 | 222 | 222 | 222 |
| Placebo | 44 | 44 | 88 | 44 | 44 | 44 | 44 |
| LRTD = lower respiratory tract disease.  ^a^Definitions of Strata: S1 = Age 60-74, LRTD risk present, Northern Hemisphere; S2 = Age 60-74, LRTD risk present, Southern Hemisphere; S3 = Age ≥75, LRTD risk present Southern or Northern Hemisphere; S4 = Age 60-74, LRTD risk absent, Northern Hemisphere; S5= Age 60-74, LRTD risk absent, Southern Hemisphere; S6 = Age ≥75, LRTD risk absent, Northern Hemisphere; S7 = Age ≥75, LRTD risk absent, Southern Hemisphere. Southern Hemisphere includes Argentina, Australia, Chile, New Zealand, and South Africa; Northern Hemisphere includes: Bangladesh, Belgium, Canada, Colombia, Costa Rica, Finland, Germany, Mexico, Japan, Panama, Poland, Singapore, South Korea, Spain, Taiwan, United Kingdom, United States of America (including Puerto Rico). | | | | | | | |

The sampling and maintenance of the Random SubCohort were performed by unblinded biostatisticians and study biomarker representatives. Unblinded biostatisticians extracted the stratified Random SubCohort sample of study participants and provided the lists of Random SubCohort in blinded and unblinded manner to selected study team personnel. Study biomarker representatives received the lists of participants in the Random SubCohort list in blinded fashion.

***Statistical analysis of immunogenicity***

The immunogenicity endpoints were analyzed as follows:

• GMT for RSV-A and RSV-B nAbs and GMC for RSV bAbs (preF) with corresponding 95% CI. The 95% CIs were calculated based on the t-distribution of the log-transformed values, then back transformed to the original scale for presentation. For each Ab analysis, also included were the number of participants (n) and the median, minimum, and maximum Ab values. GMT or GMC with 95% CI were plotted at Baseline (Day 1) and Day 29 by study vaccination group.

•GMFR of RSV-A and RSV-B nAbs, and RSV bAbs (preF) with corresponding 95% CI is provided at each post-Baseline timepoint over Baseline at Day 1. The 95% CIs were calculated based on the t-distribution of the log transformed values, then back transformed to the original scale for presentation. GMFR and corresponding 95% CI were plotted at each timepoint.

• SRR (post-injection titer of ≥4× the LLOQ if baseline was <LLOQ or ≥4-fold increase from Baseline in post-injection titers if baseline was ≥LLOQ) for RSV-A and RSV-B nAbs and RSV bAbs at each post-Baseline timepoint is provided with a 2-sided 95% CIs using the Clopper-Pearson method by study vaccination group.

• Proportion of participants with ≥2-fold increases from Baseline in RSV-A nAb titers, RSV-B nAb titers, and RSV bAbs (preF) concentration at each post-Baseline timepoint is provided with 2-sided 95% CIs using the Clopper-Pearson method by study vaccination group.

The above specified immunogenicity analyses were also performed by the following subgroups including Age group 1 (≥60 and <75 years vs. ≥75 years), age group 2 (≥60 and <70 years, ≥70 and <80 years, or ≥80 years), gender (female vs. male), Race (White, Black, Asian, or Other [including American Indian or Alaska Native, Native Hawaiian or other Pacific Islander and other races]), ethnicity (Hispanic or Latino vs. Not Hispanic or Latino), risk factors for LRTD (CHF/COPD absent vs. present), comorbidities of interest (0 and ≥1 pre-existing comorbidities of interest), frailty (based on the Edmonton Frail Scale), and World bank region (North America/Europe, Central/Latin America/Africa, or Asia Pacific).[1]

## ***Immunogenicity Assays***

*Microneutralization Assay*

Serum neutralizing antibody titers against RSV-A (RSV-A2; VR 1540 from American Type Culture Collection [ATCC], Manassas, VA) and RSV-B (RSV B 18537; VR1580, ATCC) were quantitatively measured using a validated microneutralization assay. A constant amount of RSV was mixed with serial dilutions of human sera. If RSV-specific nAb were present, RSV was neutralized and virus propagation in HEp-2 cells was inhibited. Following an incubation period, cells were fixed and immunostained with a murine monoclonal antibody directed against RSV F protein, followed by horseradish peroxidase-conjugated goat-anti-mouse antibody and TrueBlue™ (TB) substrate. The plates were scanned with a UV Analyzer and spot counts/well at each serum/antibody concentration were quantified. These values were used to determine the dilution of serum antibody that corresponds to the 50% reduction point. Results are expressed as International Units/mL (IU/ml). Conversion of nAb titers to IU/mL was performed by multiplying the sample’s absolute titer by 1.0578 for RSV-A or 0.6936 for RSV-B, as per the international standard antiserum to RSV-A and RSV-B from the World Health Organization (National Institute for Biological Standards and Control; https://www.nibsc.org).

*Binding Antibody Assay*

Serum IgG bAb against RSV preF antigen was measured using a validated quantitative multiplex assay based on Luminex® technology in which antigen-specific serum antibodies bind directly to the epitopes on antigens covalently conjugated to microspheres. The measured signal was directly proportional to the amount of serum IgG antibodies specific for RSV preF present in the serum samples. Results are expressed in arbitrary units per mL (AU/mL).

# **Figure S1. Distribution of RSV-A and RSV-B Neutralizing Antibody Titers in the Per-Protocol Immunogenicity Set**

1. **RSV-A**

1. **RSV-B**

**Figure S1**. GMT = Geometric Mean Titers; IU=International Units; n = Number of participants with non-missing data at the visit (baseline and post-baseline; n=332 for Placebo RSV-A and Placebo-RSV-B; n=1511 for mRNA-1345 RSV-A; n=1509 for mRNA-1345 RSV-B); RSV=Respiratory Syncytial Virus. Distribution of neutralizing antibody titers (log_10_ [IU/mL]) in the microneutralization assay against RSV-A and RSV-B are shown for serum samples collected before the injection of 50-µg of mRNA-1345 or placebo (baseline), and at 29 days (Day 29) after the injection in the Per-protocol Immunogenicity Set. The circles are titers from individual serum samples. The lower limits of quantification (LLOQ) in the microneutralization neutralizing antibody assay are 13 (1.1 log_10_) for RSV-A and 10 (1.0 log_10_) for RSV-B; Upper limits of quantification (ULOQ) are 259,061 (5.41 log_10_) for RSV-A and 112,476 (5.05 log_10_) for RSV-B. Boxes and horizontal bars denote interquartile ranges (IQR) and median endpoint titers; whisker endpoints are the maximum and minimum values below or above the median ±1.5 times the IQR. The brackets show GM Fold-rises which were determined for GM Titers at Day 29 vs. baseline.

# **Table S1. Stratification for Random Immunogenicity SubCohort Sampling for Per-protocol Immunogenicity Set**

|  | **Placebo (N=333) n (%)** | **mRNA-1345 50 µg (N=1515) n (%)** | **Total (N=1848) n (%)** |
| --- | --- | --- | --- |
| Age at Randomization^a^ |  |  |  |
| 60 to 74 Years | 168 (50.5) | 832 (54.9) | 1000 (54.1) |
| ≥ 75 Years | 165 (49.5) | 683 (45.1) | 848 (45.9) |
| LRTD Risk at Randomization^a^ |  |  |  |
| Present | 166 (49.8) | 659 (43.5) | 825 (44.6) |
| Absent | 167 (50.2) | 856 (56.5) | 1023 (55.4) |
| Region |  |  |  |
| Northern Hemisphere | 195 (58.6) | 848 (56.0) | 1043 (56.4) |
| Southern Hemisphere | 138 (41.4) | 667 (44.0) | 805 (43.6) |
| Stratum for Random Sampling^a^ |  |  |  |
| Age 60 to 74 Years, LRTD Risk Present, Northern Hemisphere | 43 (12.9) | 217 (14.3) | 260 (14.1) |
| Age 60 to 74 Years, LRTD Risk Present, Southern Hemisphere | 39 (11.7) | 188 (12.4) | 227 (12.3) |
| Age ≥ 75 Years, LRTD Risk Present | 84 (25.2) | 254 (16.8) | 338 (18.3) |
| Age 60 to 74 Years, LRTD Risk Absent, Northern Hemisphere | 43 (12.9) | 215 (14.2) | 258 (14.0) |
| Age 60 to 74 Years, LRTD Risk Absent, Southern Hemisphere | 43 (12.9) | 212 (14.0) | 255 (13.8) |
| Age ≥ 75 Years, LRTD Risk Absent, Northern Hemisphere | 43 (12.9) | 217 (14.3) | 260 (14.1) |
| Age ≥ 75 Years, LRTD Risk Absent, Southern Hemisphere | 38 (11.4) | 212 (14.0) | 250 (13.5) |
| LRTD=Lower Respiratory Tract Disease.  The Per-protocol Immunogenicity (PPI) set consisted of a randomly selected subset of participants (i.e. participants in the Random Subcohort) who a) received the assigned IP dose according to protocol, and b) had RSV immunogenicity titer results at baseline (prior to the study vaccine administration) and at least 1 valid result after the study vaccine administration at timepoint of interest, and c) have no major protocol deviation affecting the primary immunogenicity outcomes as determined prior to database lock and unblinding.  Percentages are based on the number of participants in the Per-Protocol Immunogenicity Set.  ^a^ Age (60 to 74 years or ≥ 75 years) and LRTD risk (Present or Absent) are based on IRT (Interactive Response Technology). | | | |

# **Table S2. Summary of RSV-A and RSV-B Neutralizing Antibody Titers in Seroresponders and Non-seroresponders at Day 29​**

|  | **RSV-A (N=1515)** | | **RSV-B (N=1515)** | |
| --- | --- | --- | --- | --- |
|  | **Seroresponders**  **(N=1119)** | **Non-seroresponders**  **(N=396)** | **Seroresponders**  **(N=851)** | **Non-seroresponders**  **(N=664)** |
| Baseline Day 1, n | 1119 | 394 | 851 | 661 |
| GMT (IU/mL) | 2006.0 | 5062.5 | 1038.7 | 2142.1 |
| (95% CI)^a^ | (1898.8-2119.3) | (4469.6-5734.1) | (977.6-1103.6) | (1975.6-2322.7) |
| Range (minimum, maximum) | 175-59460 | 249-259,061 | 94-17,960 | 164-112,476 |
| Day 29, n | 1119 | 392 | 851 | 658 |
| GMT (IU/mL) | 27,165.5 | 10,978.8 | 10197.7 | 4657.6 |
| (95% CI)^a^ | (25,581.2-28,847.8) | (9755.0-12356.1) | (9544.9-10895.2) | (4309.2-5034.2) |
| Range (minimum, maximum) | 1,402-259,061 | 512-259,061 | 454-112,476 | 122-112,476 |
| GM Fold-rise (95% CI)^a^ | 13.5 (12.9-14.2) | 2.2 (2.1-2.3) | 9.8 (9.4-10.3) | 2.2 (2.1-2.3) |
| Seroresponse rate, n/N1 (%)^b^ | 1119/1119 (100.0) | 0/390 (0.0) | 851/851 (100.0) | 0/655 (0.0) |
| (95% CI)^c^ | (99.7-100.0) | (0.0-0.9) | (99.6-100.0) | (0.0-0.6) |
| ≥2-fold increase from baseline, n/N1 (%)^d^ | 1119/1119 (100.0) | 260/390 (66.7) | 851/851 (100.0) | 418/655 (63.8) |
| (95% CI)^c^ | (99.7-100.0) | (61.7-71.3) | (99.6-100.0) | (60.0-67.5) |
| CI= Confidence Interval; Geometric mean =GM GMT=GM titer; GM Fold rise= GMTs at day 29 vs. baseline; IU=International Units; N=number of participants in the Per-Protocol Immunogenicity Set; n= Number of participants with non-missing data at the visit (baseline or post-baseline); N1= Number of participants with non-missing data at baseline and the corresponding post-baseline visit; RSV=Respiratory Syncytial Virus; RSV-A and RSV-B=RSV A and B subtypes. GMT (IU/mL) of neutralizing antibodies for RSV-A and RSV-B in Seroresponders and Non-seroresponders in the Per-Protocol Immunogenicity Set were determined in microneutralization assays. Antibody values reported as below the lower limit of quantification (LLOQ; 13 for RSV-A; 10 for RSV-B) were replaced by 0.5 x LLOQ. Values greater than the upper limit of quantification (ULOQ; 259,061 for RSV-A; 112,476 for RSV-B) are replaced by the ULOQ.  ^a^95% CI was calculated based on the t-distribution of the log-transformed values or the difference in the log-transformed values for GM value and GM fold-rise, respectively, then back transformed to the original scale for presentation.  ^b^Seroresponse at a participant level is defined as a change from below the LLOQ to equal or above 4 x LLOQ, or at least a 4-fold increase if baseline is equal to or above the LLOQ. Number of participants meeting the criterion at the time point. Percentages were based on N1.  ^c^95% CI was calculated using the Clopper-Pearson method.  ^d^≥2-fold increase in GMT at day 29 from baseline at participant level was defined as a change from below the LLOQ to equal or above 2 x LLOQ, or at least a 2-fold increase if baseline was equal to or above the LLOQ. Number of participants meeting the criterion at the time point. Percentages were based on N1. | | | | |

# **Table S3. RSV-A and RSV-B Neutralizing Antibody Titers at Day 15 (Phase 2) in the Per-Protocol Immunogenicity Set by Microneutralization Assay**

|  | **RSV-A** | | **RSV-B** | |
| --- | --- | --- | --- | --- |
|  | **mRNA-1345 50 µg**  **(N=1515)** | **Placebo**  **(N=333)** | **mRNA-1345 50 µg**  **(N=1515)** | **Placebo**  **(N=333)** |
| Baseline Day 1, n | 1513 | 333 | 1512 | 333 |
| GMT (IU/mL) | 2,552.8 | 2,403.7 | 1,425.4 | 1,350.3 |
| (95% CI)^a^ | (2,414.3-2,699.4) | (2,136.0-2,705.0) | (1,352.7-1,501.9) | (1,203.3-1,515.2) |
| Range (minimum, maximum) | 175-259,061 | 157-106,190 | 94-112,476 | 114-79,619 |
| Day 15, n | 68 | 20 | 68 | 20 |
| GMT (IU/mL) | 28,952.0 | 2,392.2 | 10,311.0 | 1,481.0 |
| (95% CI)^a^ | (21,793.8-38,461.3) | (1,378.7-4,150.8) | (7,933.0-13,401.9) | (1,019.7-2,151.2) |
| Range (minimum, maximum) | 521-259,061 | 219-23,431 | 1,188-84,579 | 240-9,487 |
| GM Fold-rise (95% CI) ^a^ | 9.2 (7.0-12.1) | 0.9 (0.7-1.2) | 6.4 (4.9-8.2) | 1.2 (1.0-1.4) |
| Seroresponse rate, n/N1 (%)^b^ | 53/68 (77.9) | 0/20 (0.0) | 46/68 (67.6) | 0/20 (0.0) |
| (95% CI)^c^ | (66.2-87.1) | (0.0-16.8) | (55.2-78.5) | (0.0-16.8) |
| ≥2-fold increase from baseline, n/N1 (%)^d^ | 63/68 (92.6) | 1/20 (5.0) | 57/68 (83.8) | 2/20 (10.0) |
| (95% CI) ^c^ | (83.7-97.6) | (0.1-24.9) | (72.9-91.6) | (1.2-31.7) |
| CI= Confidence Interval; GM=Geometric Mean; GMT=GM titer; IU=International Units; N=number of participants in the Per-protocol Immunogenicity Set; n= Number of participants with non-missing data at the visit (baseline or post-baseline); N1= Number of participants with non-missing data at baseline and the corresponding post-baseline visit; RSV=Respiratory Syncytial Virus; RSV-A and RSV-B=RSV A and B subtypes. GMT (IU/mL) of neutralizing antibodies for RSV-A and RSV-B in participants in the Per-protocol Immunogenicity Set were determined in microneutralization assays. Antibody values reported as below the lower limit of quantification (LLOQ; 13 for RSV-A; 10 for RSV-B) were replaced by 0.5 x LLOQ. Values greater than the upper limit of quantification (ULOQ; 259,061 for RSV-A; 112,476 for RSV-B) were replaced by the ULOQ.  ^a^ 95% CI was calculated based on the t-distribution of the log-transformed values or the difference in the log-transformed values for GM value and GM fold-rise, respectively, then back transformed to the original scale for presentation.  ^b^ Seroresponse at a participant level was defined as a change from below the LLOQ to equal or above 4 x LLOQ, or at least a 4-fold increase if baseline was equal to or above the LLOQ. Number of participants meeting the criterion at the time point. Percentages were based on N1.  ^c^ 95% CI was calculated using the Clopper-Pearson method.  ^d^ ≥2-fold increase in GMT at day 29 from baseline at participant level was defined as a change from below the LLOQ to equal or above 2 x LLOQ, or at least a 2-fold increase if baseline is equal to or above the LLOQ. Number of participants meeting the criterion at the time point. Percentages were based on N1. | | | | |

# **Table S4. RSV-A Neutralizing Antibody Titers and GMFRs at Day 29 Following mRNA-1345 by Subgroups in the Per-Protocol Immunogenicity Set ​**

|  | **Day 1 GMT** | | **Day 29 GMT** | | **Day 29 vs Day 1** | | **SRR^c^** | | **≥2-fold increase from baseline^f^** | |
| --- | --- | --- | --- | --- | --- | --- | --- | --- | --- | --- |
|  | **n^a^** | **GMT (IU/mL)** **(95% CI)^b^** | **n^a^** | **GMT (IU/mL)** **(95% CI)^b^** | **N1^a^** | **GMFR** **(95% CI)^b^** | **n/N1^d^** | **% (95% CI)^e^** | **n/N1^d^** | **% (95% CI)^e^** |
| Overall | 1513 | 2552.8 (2414.3-2699.4) | 1511 | 21475.4 (20273.9-22748.1) | 1509 | 8.4 (8.0-8.9) | 1119/1509 | 74.2 (71.9-76.3) | 1379/1509 | 91.4 (89.9-92.8) |
| CHF/COPD^g^ |  |  |  |  |  |  |  |  |  |  |
| Absent | 928 | 2358.3 (2199.7-2528.3) | 928 | 19918.7 (18506.8-21438.3) | 926 | 8.5 (7.9-9.1) | 684/926 | 73.9 (70.9-76.7) | 856/926 | 92.4 (90.5-94.1) |
| Present | 585 | 2894.5 (2639.5-3174.9) | 583 | 24208.1 (22080.3-26540.9) | 583 | 8.4 (7.6-9.2) | 435/583 | 74.6 (70.9-78.1) | 523/583 | 89.7 (87.0-92.1) |
| Age Group^g^ |  |  |  |  |  |  |  |  |  |  |
| 60-74 years | 835 | 2437.8 (2267.9-2620.4) | 835 | 22492.0 (20887.1-24220.1) | 834 | 9.2 (8.6-9.9) | 652/834 | 78.2 (75.2-80.9) | 773/834 | 92.7 (90.7, 94.4) |
| ≥75 years | 678 | 2702.0 (2476.4-2948.2) | 676 | 20282.9 (18526.6-22205.8) | 675 | 7.6 (7.0-8.2) | 467/675 | 69.2 (65.5-72.7) | 606/675 | 89.8 (87.2-92.0) |
| Age by Decade^g^ |  |  |  |  |  |  |  |  |  |  |
| 60-69 years | 619 | 2309.0 (2123.5-2510.7) | 618 | 22611.8 (20733.8-24659.9) | 618 | 9.8 (9.0-10.7) | 492/618 | 79.6 (76.2-82.7) | 583/618 | 94.3 (92.2-96.0) |
| 70-79 years | 671 | 2746.8 (2517.8-2996.6) | 671 | 20374.3 (18646.9-22261.7) | 669 | 7.5 (6.9-8.1) | 474/669 | 70.9 (67.2-74.3) | 592/669 | 88.5 (85.8-90.8) |
| ≥80 years | 223 | 2706.1 (2344.6-3123.4) | 222 | 21811.7 (18680.7-25467.6) | 222 | 8.1 (7.0-9.4) | 153/222 | 68.9 (62.4-74.9) | 204/222 | 91.9 (87.5-95.1) |
| Comorbidities of Interest^h^ |  |  |  |  |  |  |  |  |  |  |
| Zero | 647 | 2410.1 (2216.2-2621.0) | 647 | 19270.1 (17641.7-21048.9) | 646 | 8.0 (7.4-8.7) | 462/646 | 71.5 (67.9-75.0) | 595/646 | 92.1 (89.8-94.1) |
| ≥1 | 866 | 2665.0 (2473.1-2871.7) | 864 | 23290.5 (21594.0-25120.3) | 863 | 8.8 (8.1-9.5) | 657/863 | 76.1 (73.1-78.9) | 784/863 | 90.8 (88.7-92.7) |
| Frailty Status 1^g^ |  |  |  |  |  |  |  |  |  |  |
| Fit (0-3) | 1032 | 2520.9 (2358.6-2694.4) | 1032 | 20826.4 (19428.4-22325.0) | 1030 | 8.3 (7.8-8.9) | 766/1030 | 74.4 (71.6-77.0) | 947/1030 | 91.9 (90.1-93.5) |
| Vulnerable (4-5) | 310 | 2461.5 (2177.2-2782.9) | 310 | 21972.1 (19283.2-25036.1) | 310 | 8.9 (7.8-10.2) | 228/310 | 73.5 (68.3-78.4) | 281/310 | 90.6 (86.8-93.6) |
| Frail (≥6) | 149 | 2900.0 (2367.8-3551.8) | 147 | 24951.5 (20768.1-29977.5) | 147 | 8.6 (7.2-10.4) | 108/147 | 73.5 (65.6-80.4) | 132/147 | 89.8 (83.7-94.2) |
| Frailty Status 2^g^ |  |  |  |  |  |  |  |  |  |  |
| Fit (0-3) | 1032 | 2520.9 (2358.6-2694.4) | 1032 | 20826.4 (19428.4-22325.0) | 1030 | 8.3 (7.8-8.9) | 766/1030 | 74.4 (71.6-77.0) | 947/1030 | 91.9 (90.1-93.5) |
| Vulnerable/Frail (≥4) | 459 | 2596.0 (2335.8-2885.3) | 457 | 22889.5 (20583.8-25453.4) | 457 | 8.8 (7.9-9.8) | 336/457 | 73.5 (69.2-77.5) | 413/457 | 90.4 (87.3-92.9) |
| Gender |  |  |  |  |  |  |  |  |  |  |
| Male | 833 | 2774.0 (2570.3-2993.8) | 831 | 20988.8 (19385.9-22724.1) | 831 | 7.6 (7.0-8.2) | 583/831 | 70.2 (66.9-73.3) | 738/831 | 88.8 (86.5-90.9) |
| Female | 680 | 2305.8 (2125.6-2501.3) | 680 | 22085.4 (20316.5-24008.4) | 678 | 9.6 (8.9-10.4) | 536/678 | 79.1 (75.8-82.1) | 641/678 | 94.5 (92.6-96.1) |
| Race Group |  |  |  |  |  |  |  |  |  |  |
| White | 1170 | 2598.0 (2436.2-2770.4) | 1169 | 21641.8 (20276.5-23099.0) | 1167 | 8.4 (7.9-8.9) | 862/1167 | 73.9 (71.2-76.4) | 1066/1167 | 91.3 (89.6-92.9) |
| Black | 133 | 2409.5 (1966.8-2951.9) | 132 | 21123.9 (17058.2-26158.5) | 132 | 8.8 (7.3-10.5) | 103/132 | 78.0 (70.0-84.8) | 119/132 | 90.2 (83.7-94.7) |
| Asian | 66 | 2548.9 (2001.6-3245.8) | 66 | 19071.2 (14629.4-24861.5) | 66 | 7.5 (6.0-9.4) | 46/66 | 69.7 (57.1-80.4) | 61/66 | 92.4 (83.2-97.5) |
| Other^i^ | 141 | 2345.0 (1992.6-2759.7) | 141 | 20881.2 (17381.8-25085.1) | 141 | 8.9 (7.4-10.7) | 105/141 | 74.5 (66.4-81.4) | 130/141 | 92.2 (86.5-96.0) |
| World Bank Region |  |  |  |  |  |  |  |  |  |  |
| North America/Europe | 684 | 2482.1 (2278.8-2703.5) | 683 | 20681.2 (18910.3-22617.9) | 681 | 8.4 (7.7-9.1) | 495/681 | 72.7 (69.2-76.0) | 615/681 | 90.3 (87.8-92.4) |
| Central/Latin America/Africa | 721 | 2625.2 (2424.8-2842.2) | 721 | 22957.5 (21199.8-24860.9) | 721 | 8.8 (8.1-9.5) | 553/721 | 76.7 (73.4-79.7) | 670/721 | 92.9 (90.8-94.7) |
| Asia Pacific | 108 | 2530.8 (2074.2-3087.9) | 107 | 17422.5 (14046.5-21610.0) | 107 | 7.0 (5.7-8.5) | 71/107 | 66.4 (56.6-75.2) | 94/107 | 87.9 (80.1-93.4) |
| Region |  |  |  |  |  |  |  |  |  |  |
| USA | 573 | 2667.5 (2424.0-2935.4) | 572 | 21093.0 (19114.0-23276.8) | 570 | 8.0 (7.2-8.8) | 405/570 | 71.1 (67.1-74.7) | 508/570 | 89.1 (86.3-91.6) |
| Non-USA | 940 | 2485.4 (2321.1-2661.3) | 939 | 21711.7 (20229.5-23302.6) | 939 | 8.8 (8.2-9.4) | 714/939 | 76.0 (73.2-78.7) | 871/939 | 92.8 (90.9-94.3) |
| Ethnicity |  |  |  |  |  |  |  |  |  |  |
| Hispanic or Latino | 705 | 2604.3 (2401.7-2824.2) | 705 | 22389.5 (20680.1-24240.1) | 705 | 8.6 (7.9-9.3) | 534/705 | 75.7 (72.4-78.9) | 651/705 | 92.3 (90.1-94.2) |
| Non-Hispanic or Latino | 787 | 2488.2 (2300.7-2690.9) | 785 | 20410.9 (18781.9-22181.2) | 783 | 8.3 (7.6-8.9) | 570/783 | 72.8 (69.5-75.9) | 711/783 | 90.8 (88.6-92.7) |
| CI = Confidence Interval; CHF=Congestive Heart Failure; COPD=Chronic Obstructive Pulmonary Disease;; GM = Geometric Mean; GMFR=geometric mean fold-rise; GMT =GM titer; LRTD=Lower Respiratory Tract Disease; SRR=seroresponse; N1 = Number of participants with non-missing data at baseline and the corresponding post-baseline visit. GMTs (IU/mL) of neutralizing antibodies for RSV-A in participants in the Per-Protocol Immunogenicity Set were determined in microneutralization assays. Antibody values reported as below the lower limit of quantification (LLOQ; 13 for RSV-A) were replaced by 0.5 x LLOQ. Values greater than the upper limit of quantification (ULOQ; 259,061 for RSV-A) were replaced by the ULOQ. GM Fold-rises were determined for GMTs at Day 29 vs. baseline.  ^a^Number of participants in the Per-Protocol Immunogenicity Set with non-missing data at the visit (baseline or post-baseline).  ^b^95% CI was calculated based on the t-distribution of the log-transformed values or the difference in the log-transformed values for GM value and GM fold-rise, respectively, then back transformed to the original scale for presentation.  ^c^Seroresponse at a participant level is defined as a change from below the LLOQ to equal or above 4 x LLOQ, or at least a 4-fold increase if baseline is equal to or above the LLOQ.  ^d^Number of participants meeting the criterion at the time point. Percentages are based on N1.  ^e^95% CI is calculated using the Clopper-Pearson method.  ^f^2-fold increase in GMT at day 29 from baseline at participant level was defined as a change from below the LLOQ to equal or above 2 x LLOQ, or at least a 2-fold increase if baseline was equal to or above the LLOQ.  ^g^Derived from age and risk factors collected on electronic case report forms. Assignment to vaccination groups was stratified by age (60 to 74 years versus ≥ 75 years) and risk factors for LRTD (present versus absent). Baseline value for Edmonton Frail Scale total score was defined as the most recent non-missing measurement (scheduled or unscheduled) collected on or before the date of injection of mRNA-1345 or placebo.  ^h^Comorbidities of Interest include COPD, asthma, chronic respiratory disease, diabetes, CHF, advanced liver disease or advanced renal disease.  ^i^Other race includes American Indian or Alaska Native, Native Hawaiian or other Pacific Islander, Other, or Multiple. | | | | | | | | | | |

# **Table S5. RSV-B Neutralizing Antibody Titers and GMFRs at Day 29 Following mRNA-1345 by Subgroups in the Per-Protocol Immunogenicity Set**

​

|  | **Day 1 GMT** | | **Day 29 GMT** | | **Day 29 vs Day 1** | | **SRR^c^** | | **≥2-fold increase from baseline^f^** | |
| --- | --- | --- | --- | --- | --- | --- | --- | --- | --- | --- |
|  | **n^a^** | **GMT (IU/mL)** **(95% CI)^b^** | **n^a^** | **GMT (IU/mL)** **(95% CI)^b^** | **N1^a^** | **GMFR** **(95% CI)^b^** | **n/N1^d^** | **% (95% CI)^e^** | **n/N1^d^** | **% (95% CI)^e^** |
| Overall | 1512 | 1425.4 (1352.7-1501.9) | 1509 | 7246.0 (6864.8-7648.4) | 1506 | 5.1 (4.9-5.4) | 851/1506 | 56.5 (54.0-59.0) | 1269/1506 | 84.3 (82.3-86.1) |
| CHF/COPD^g^ |  |  |  |  |  |  |  |  |  |  |
| Absent | 928 | 1325.6 (1243.0-1413.7) | 926 | 6801.4 (6345.6-7290.0) | 924 | 5.2 (4.8-5.5) | 526/924 | 56.9 (53.7-60.1) | 779/924 | 84.3 (81.8-86.6) |
| Present | 584 | 1599.5 (1464.4-1747.2) | 583 | 8012.6 (7353.6-8730.6) | 582 | 5.1 (4.7-5.5) | 325/582 | 55.8 (51.7-59.9) | 490/582 | 84.2 (81.0-87.1) |
| Age Group^g^ |  |  |  |  |  |  |  |  |  |  |
| 60-74 years | 834 | 1430.1 (1331.0-1536.7) | 833 | 7757.3 (7215.2-8340.2) | 831 | 5.5 (5.1-5.8) | 480/831 | 57.8 (54.3-61.1) | 717/831 | 86.3 (83.8-88.5) |
| ≥75 years | 678 | 1419.5 (1315.1-1532.2) | 676 | 6662.0 (6144.3-7223.4) | 675 | 4.7 (4.4-5.1) | 371/675 | 55.0 (51.1- 58.8) | 552/675 | 81.8 (78.7-84.6) |
| Age by Decade^g^ |  |  |  |  |  |  |  |  |  |  |
| 60-69 years | 617 | 1353.2 (1247.9-1467.3) | 616 | 7456.8 (6856.6-8109.5) | 614 | 5.5 (5.1-6.0) | 355/614 | 57.8 (53.8-61.8) | 533/614 | 86.8 (83.9-89.4) |
| 70-79 years | 672 | 1498.2 (1382.0-1624.2) | 671 | 7092.5 (6531.9-7701.2) | 670 | 4.8 (4.4-5.1) | 368/670 | 54.9 (51.1-58.7) | 547/670 | 81.6 (78.5-84.5) |
| ≥80 years | 223 | 1416.2 (1244.4-1611.8) | 222 | 7139.0 (6211.8-8204.6) | 222 | 5.1 (4.5-5.7) | 128/222 | 57.7 (50.9-64.2) | 189/222 | 85.1 (79.8-89.5) |
| Comorbidities of Interest^h^ |  |  |  |  |  |  |  |  |  |  |
| Zero | 647 | 1317.8 (1220.2-1423.2) | 645 | 6474.2 (5963.0-7029.2) | 644 | 4.9 (4.6-5.3) | 345/644 | 53.6 (49.6-57.5) | 538/644 | 83.5 (80.4-86.3) |
| ≥1 | 865 | 1511.5 (1408.0-1622.6) | 864 | 7881.6 (7339.0-8464.2) | 862 | 5.3 (4.9-5.6) | 506/862 | 58.7 (55.3-62.0) | 731/862 | 84.8 (82.2-87.1) |
| Frailty Status 1^g^ |  |  |  |  |  |  |  |  |  |  |
| Fit (0-3) | 1032 | 1414.6 (1326.8-1508.2) | 1029 | 6937.2 (6491.8-7413.1) | 1027 | 4.9 (4.7-5.2) | 562/1027 | 54.7 (51.6-57.8) | 856/1027 | 83.3 (80.9-85.6) |
| Vulnerable (4-5) | 309 | 1404.0 (1255.9-1569.7) | 310 | 7680.4 (6831.3-8635.1) | 309 | 5.5 (4.9-6.2) | 181/309 | 58.6 (52.9-64.1) | 266/309 | 86.1 (81.7-89.7) |
| Frail (≥6) | 149 | 1568.4 (1319.7-1864.0) | 148 | 8730.4 (7368.9-10343.4) | 148 | 5.6 (4.7-6.5) | 92/148 | 62.2 (53.8-70.0) | 125/148 | 84.5 (77.6-89.9) |
| Frailty Status 2^g^ |  |  |  |  |  |  |  |  |  |  |
| Fit (0-3) | 1032 | 1414.6 (1326.8-1508.2) | 1029 | 6937.2 (6491.8-7413.1) | 1027 | 4.9 (4.7-5.2) | 562/1027 | 54.7 (51.6-57.8) | 856/1027 | 83.3 (80.9-85.6) |
| Vulnerable/Frail (≥4) | 458 | 1455.5 (1325.4-1598.4) | 458 | 8005.1 (7271.0-8813.3) | 457 | 5.5 (5.0-6.1) | 273/457 | 59.7 (55.1-64.3) | 391/457 | 85.6 (82.0-88.7) |
| Gender |  |  |  |  |  |  |  |  |  |  |
| Male | 831 | 1487.1 (1383.8-1598.2) | 831 | 7170.2 (6651.8-7729.0) | 829 | 4.8 (4.5-5.2) | 454/829 | 54.8 (51.3-58.2) | 674/829 | 81.3 (78.5-83.9) |
| Female | 681 | 1353.4 (1254.4-1460.3) | 678 | 7340.0 (6791.2-7933.2) | 677 | 5.5 (5.1-5.9) | 397/677 | 58.6 (54.8-62.4) | 595/677 | 87.9 (85.2-90.2) |
| Race Group |  |  |  |  |  |  |  |  |  |  |
| White | 1169 | 1458.4 (1374.6-1547.3) | 1168 | 7329.2 (6896.0-7789.7) | 1165 | 5.1 (4.8-5.3) | 661/1165 | 56.7 (53.8-59.6) | 977/1165 | 83.9 (81.6-85.9) |
| Black | 133 | 1263.7 (1028.7-1552.4) | 132 | 6702.8 (5498.6-8170.8) | 132 | 5.3 (4.4-6.4) | 71/132 | 53.8 (44.9-62.5) | 110/132 | 83.3 (75.9-89.3) |
| Asian | 66 | 1188.6 (924.9-1527.5) | 65 | 5775.0 (4495.3-7419.1) | 65 | 4.9 (4.0-6.1) | 34/65 | 52.3 (39.5-64.9) | 58/65 | 89.2 (79.1-95.6) |
| Other^i^ | 141 | 1453.1 (1244.8-1696.4) | 141 | 7631.4 (6379.8-9128.5) | 141 | 5.3 (4.5-6.2) | 82/141 | 58.2 (49.6-66.4) | 121/141 | 85.8 (78.9-91.1) |
| World Bank Region |  |  |  |  |  |  |  |  |  |  |
| North America/Europe | 683 | 1253.6 (1155.7-1359.7) | 683 | 6377.9 (5871.2-6928.3) | 680 | 5.1 (4.8-5.5) | 387/680 | 56.9 (53.1-60.7) | 567/680 | 83.4 (80.4-86.1) |
| Central/Latin America/Africa | 721 | 1656.7 (1543.0-1778.7) | 720 | 8476.4 (7865.3-9135.0) | 720 | 5.1 (4.8-5.5) | 401/720 | 55.7 (52.0-59.4) | 610/720 | 84.7 (81.9-87.3) |
| Asia Pacific | 108 | 1176.6 (965.1-1434.3) | 106 | 5682.4 (4655.6-6935.6) | 106 | 5.0 (4.2-6.0) | 63/106 | 59.4 (49.5-68.9) | 92/106 | 86.8 (78.8-92.6) |
| Region |  |  |  |  |  |  |  |  |  |  |
| USA | 572 | 1313.6 (1200.4-1437.4) | 572 | 6411.5 (5852.3-7024.1) | 569 | 4.9 (4.5-5.3) | 310/569 | 54.5 (50.3-58.6) | 467/569 | 82.1 (78.7-85.1) |
| Non-USA | 940 | 1498.0 (1405.5-1596.6) | 937 | 7808.0 (7305.2-8345.3) | 937 | 5.2 (4.9-5.6) | 541/937 | 57.7 (54.5-60.9) | 802/937 | 85.6 (83.2-87.8) |
| Ethnicity |  |  |  |  |  |  |  |  |  |  |
| Hispanic or Latino | 704 | 1688.3 (1570.3-1815.2) | 705 | 8554.0 (7925.5-9232.4) | 704 | 5.1 (4.7-5.5) | 392/704 | 55.7 (51.9-59.4) | 595/704 | 84.5 (81.6-87.1) |
| Non-Hispanic or Latino | 787 | 1218.5 (1131.8-1311.9) | 783 | 6164.0 (5715.6-6647.7) | 781 | 5.1 (4.8-5.5) | 446/781 | 57.1 (53.5-60.6) | 657/781 | 84.1 (81.4-86.6) |
| CI = Confidence Interval; CHF=Congestive Heart Failure; COPD=Chronic Obstructive Pulmonary Disease; GM=Geometric Mean; GMFR=geometric mean fold-rise; GMT=GM titer; LRTD=Lower Respiratory Tract Disease; SRR=seroresponse. N1=Number of participants with non-missing data at baseline and the corresponding post-baseline visit. GMTs (IU/mL) of neutralizing antibodies for RSV-B in participants in the Per-Protocol Immunogenicity Set were determined in microneutralization assays. Antibody values reported as below the lower limit of quantification (LLOQ; 10 for RSV-B) were replaced by 0.5 x LLOQ. Values greater than the upper limit of quantification (ULOQ; 112,476 for RSV-B) were replaced by the ULOQ. GM Fold-rises were determined for GMT at Day 29 vs. baseline.  ^a^Number of participants in the Per-Protocol Immunogenicity Set with non-missing data at the visit (baseline or post-baseline).  ^b^95% CI was calculated based on the t-distribution of the log-transformed values or the difference in the log-transformed values for GM value and GM fold-rise, respectively, then back transformed to the original scale for presentation.  ^c^Seroresponse at a participant level is defined as a change from below the LLOQ to equal or above 4 x LLOQ, or at least a 4-fold increase if baseline is equal to or above the LLOQ.  ^d^Number of participants meeting the criterion at the time point. Percentages are based on N1.  ^e^95% CI is calculated using the Clopper-Pearson method.  ^f^2-fold increase in GMT at day 29 from baseline at participant level was defined as a change from below the LLOQ to equal or above 2 x LLOQ, or at least a 2-fold increase if baseline was equal to or above the LLOQ.  ^g^Derived from age and risk factors collected on electronic case report forms. Assignment to vaccination groups was stratified by age (60 to 74 years versus ≥ 75 years) and risk factors for LRTD (present versus absent). Baseline value for Edmonton Frail Scale total score iwas defined as the most recent non-missing measurement (scheduled or unscheduled) collected on or before the date of injection of mRNA-1345 or placebo.  ^h^Comorbidities of Interest include COPD, asthma, chronic respiratory disease, diabetes, CHF, advanced liver disease or advanced renal disease.  ^i^Other race includes American Indian or Alaska Native, Native Hawaiian or other Pacific Islander, Other, or Multiple. | | | | | | | | | | |

# **Table S6. Summary of RSV preF Binding Antibody Concentrations in the Per-protocol Immunogenicity Set**

|  | **preF** | |
| --- | --- | --- |
|  | **mRNA-1345 50 µg (N=1515)** | **Placebo (N=333)** |
| Baseline (Day 1) |  |  |
| n^a^ | 1513 | 333 |
| GMC | 10,729.5 | 10,194.3 |
| 95% CI^b^ | (10,310.6-11,165.5) | (9,374.5-11,085.7) |
| Range (Minimum, maximum) | 798-464,148 | 1,104-91,124 |
| Day 29 |  |  |
| n | 1511 | 333 |
| GMC | 81,884.2 | 10,060.2 |
| 95% CI^b^ | (78,644.2-85,257.6) | (9,258.9-10,930.7) |
| Range (minimum, maximum) | 1,063-580,553 | 1,153-81,862 |
| N1 | 1510 | 333 |
| GMFR (95% CI) ^b^ | 7.7 (7.3-8.0) | 1.0 (1.0-1.0) |
| Seroresponse (%)^c^ |  |  |
| n/N1 (%)^d^ | 1195/1510 (79.1) | 1/333 (0.3) |
| 95% CI^e^ | (77.0-81.2) | (0.0-1.7) |
| ≥2-fold Increase from baseline^f^ |  |  |
| n/N1 (%)^d^ | 1423/1510 (94.2) | 3/333 (0.9) |
| 95% CI^e^ | (92.9-95.4) | (0.2-2.6) |
| CI=confidence interval; GM=geometric mean; GMC=GM concentration; GMFR=geometric mean fold-rise; LLOQ=lower limit of quantification; max=maximum; min=minimum; PPIS=Per-Protocol Immunogenicity Set; RSV=respiratory syncytial virus; SRR=seroresponse rate; ULOQ=upper limit of quantification. N1 = Number of participants with non-missing data at baseline and the corresponding post-baseline visit.GMC (AU/mL) of preF binding antibodies for RSV preF in participants in the Per-Protocol Immunogenicity Set were determined in Luminex assays. Antibody values reported as below the lower limit of quantification (LLOQ; 35) are replaced by 0.5 x LLOQ. Values greater than the upper limit of quantification (ULOQ; 580,553) are replaced by the ULOQ. GM Fold-rises were determined for GMC at Day 29 vs. baseline. ^a^Number of participants with non-missing data at the visit (baseline or post-baseline).  ^b^95% CI was calculated based on the t-distribution of the log-transformed values or the difference in the log-transformed values for GM value and GM fold-rise, respectively, then back transformed to the original scale for presentation.  ^c^Seroresponse at a participant level was defined as a change from below the LLOQ to equal or above 4 x LLOQ, or at least a 4-fold increase if baseline was equal to or above the LLOQ (preF LLOQ: 35 AU/mL, ULOQ: 580,553 AU/mL).  ^d^Number of participants meeting the criterion at the timepoint. Percentages are based on N1.  ^e^95% CI is calculated using the Clopper-Pearson method.  ^f^≥2-fold increase in GMC from baseline at a participant level was defined as a change from below the LLOQ to equal or above 2 x LLOQ, or at least a 2-fold increase if baseline is equal to or above the LLOQ. | | |

# **Table S7. RSV preF Binding Antibody Concentrations and GMFRs at Day 29 Following mRNA-1345 by Subgroups and Visit in the Per-Protocol Immunogenicity Set**

| **Subgroup** | **Day 1** | | **Day 29** | | **Day 29 vs Day 1** | |
| --- | --- | --- | --- | --- | --- | --- |
|  | **N^a^** | **GMC (AU/mL)****(95% CI)^b^** | **n** | **GMC (AU/mL)****(95% CI)** | **n** | **GM Fold-rise****(95% CI)** |
| Overall | 1513 | 10729.5 (10310.6-11165.5) | 1511 | 81884.2 (78644.2-85257.6) | 1510 | 7.7 (7.3-8.0) |
| CHF/COPD^c^ |  |  |  |  |  |  |
| Absent | 929 | 9883.9 (9427.4-10362.5) | 928 | 75746.7 (71989.9-79699.6) | 927 | 7.7 (7.3-8.1) |
| Present | 584 | 12226.3 (11405.4-13106.2) | 583 | 92695.7 (86844.8-98940.7) | 583 | 7.6 (7.1-8.2) |
| Age Group^c^ |  |  |  |  |  |  |
| 60-74 years | 834 | 10274.1 (9753.9-10822.1) | 834 | 86856.3 (82438.0-91511.5) | 833 | 8.5 (8.0-9.0) |
| ≥75 years | 679 | 11316.6 (10640.8-12035.3) | 677 | 76148.5 (71511.9-81085.7) | 677 | 6.8 (6.3-7.2) |
| Age by Decade^c^ |  |  |  |  |  |  |
| 60-69 years | 618 | 9986.7 (9408.9-10600.0) | 618 | 86548.1 (81459.3-91954.9) | 617 | 8.7 (8.1-9.3) |
| 70- 79 years | 672 | 11081.3 (10436.7-11765.8) | 670 | 77891.6 (73262.7-82813.0) | 670 | 7.1 (6.6-7.5) |
| ≥80 years | 223 | 11876.9 (10597.0-13311.3) | 223 | 81611.9 (72874.5-91396.9) | 223 | 6.9 (6.1-7.7) |
| Comorbidities of Interest^d^ |  |  |  |  |  |  |
| Zero | 647 | 9942.9 (9396.4-10521.2) | 647 | 73099.9 (68809.6-77657.7) | 646 | 7.4 (6.9-7.8) |
| ≥1 | 866 | 11357.5 (10748.8-12000.8) | 864 | 89146.7 (84497.2-94052.2) | 864 | 7.9 (7.4-8.3) |
| Frailty Status 1^c^ |  |  |  |  |  |  |
| Fit (0-3) | 1033 | 10381.7 (9897.1-10889.9) | 1032 | 78989.0 (75263.9-82898.5) | 1031 | 7.6 (7.3-8.0) |
| Vulnerable (4-5) | 309 | 10962.7 (10094.5-11905.5) | 309 | 83549.6 (75964.8-91891.9) | 309 | 7.6 (6.9-8.5) |
| Frail (≥6) | 149 | 12422.2 (10674.8-14455.7) | 148 | 96247.4 (84846.1-109180.8) | 148 | 7.7 (6.7-8.9) |
| Frailty Status 2^c^ |  |  |  |  |  |  |
| Fit (0-3) | 1033 | 10381.7 (9897.1-10889.9) | 1032 | 78989.0 (75263.9-82898.5) | 1031 | 7.6 (7.3-8.0) |
| Vulnerable/Frail (≥4) | 458 | 11417.6 (10600.7-12297.5) | 457 | 87466.9 (81050.6-94391.0) | 457 | 7.7 (7.0-8.3) |
| Gender |  |  |  |  |  |  |
| Male | 832 | 11622.3 (11010.3-12268.2) | 832 | 80839.7 (76587.4-85328.1) | 831 | 7.0 (6.6-7.4) |
| Female | 681 | 9731.3 (9181.5-10314.1) | 679 | 83182.4 (78271.4-88401.4) | 679 | 8.6 (8.0-9.1) |
| Race Group |  |  |  |  |  |  |
| White | 1170 | 10871.2 (10389.1-11375.6) | 1169 | 81076.2 (77466.5-84854.2) | 1168 | 7.5 (7.1-7.9) |
| Black | 133 | 10039.0 (8616.4-11696.4) | 132 | 80354.0 (70105.0-92101.4) | 132 | 8.0 (6.9-9.3) |
| Asian | 66 | 10111.4 (8372.8-12211.1) | 66 | 75200.5 (60627.3-93276.7) | 66 | 7.4 (6.3-8.8) |
| Other^e^ | 141 | 10598.2 (9442.0-11896.0) | 141 | 92505.9 (80813.5-105890.1) | 141 | 8.7 (7.6-10.0) |
| World Bank Region |  |  |  |  |  |  |
| North America/Europe | 685 | 10400.4 (9785.4-11054.0) | 683 | 74788.4 (70305.5-79557.2) | 683 | 7.2 (6.8-7.7) |
| Central/Latin America/Africa | 720 | 11004.7 (10395.0-11650.1) | 721 | 91025.7 (86061.7-96276.0) | 720 | 8.3 (7.8-8.8) |
| Asia Pacific | 108 | 11043.0 (9641.9-12647.6) | 107 | 71574.3 (61552.0-83228.4) | 107 | 6.5 (5.6-7.6) |
| Region |  |  |  |  |  |  |
| USA | 574 | 10662.2 (9958.3-11415.9) | 572 | 73614.4 (68752.3-78820.4) | 572 | 6.9 (6.4-7.4) |
| Non-USA | 939 | 10770.8 (10257.5-11309.9) | 939 | 87370.7 (83149.6-91806.0) | 938 | 8.1 (7.7-8.6) |
| Ethnicity |  |  |  |  |  |  |
| Hispanic or Latino | 704 | 11112.2 (10482.6-11779.6) | 705 | 90222.2 (85323.5-95402.0) | 704 | 8.1 (7.6-8.7) |
| Non-Hispanic or Latino | 788 | 10358.3 (9810.9-10936.2) | 785 | 74698.0 (70516.4-79127.7) | 785 | 7.2 (6.8-7.7) |
| GM=Geometric Mean; GMC=Geometric Mean Concentration; CI=Confidence Interval; LRTD=Lower Respiratory Tract Disease; CHF=Congestive Heart Failure; COPD=Chronic Obstructive Pulmonary Disease; COVID-19=Coronavirus Disease 2019. GMC (AU/mL) of binding antibodies for RSV preF in participants in the Per-Protocol Immunogenicity Set were determined in Luminex assays. Antibody values reported as below the lower limit of quantification (LLOQ; 35) were replaced by 0.5 x LLOQ. Values greater than the upper limit of quantification (ULOQ; 580,553) were replaced by the ULOQ. GM Fold-rises were determined for GMC at Day 29 vs. baseline.^a^n=Number of participants in the Per-Protocol Immunogenicity Set with non-missing data at the visit (baseline or post-baseline); CI = Confidence Interval; GMC = Geometric Mean; AU/mL =absorbance unites per mL^b^95% CI was calculated based on the t-distribution of the log-transformed values or the difference in the log-transformed values for GM value and GM fold-rise, respectively, then back transformed to the original scale for presentation.^c^Derived from age and risk factors collected on electronic case report forms. Assignment to vaccination groups was stratified by age (60 to 74 years versus ≥ 75 years) and risk factors for LRTD (present versus absent). Baseline value for Edmonton Frail Scale total score was defined as the most recent non-missing measurement (scheduled or unscheduled) collected on or before the date of injection of mRNA-1345 or placebo.^d^Comorbidities of Interest include COPD, asthma, chronic respiratory disease, diabetes, CHF, advanced liver disease or advanced renal disease.^e^Other race includes American Indian or Alaska Native, Native Hawaiian or other Pacific Islander, Other, or Multiple. | | | | | | |

**Supplement References**

1. Rolfson DB, Majumdar SR, Tsuyuki RT, Tahir A, Rockwood K. Validity and reliability of the Edmonton Frail Scale. Age Ageing **2006**; 35:526-9.

2. Wilson E, Goswami J, Baqui AH, et al. Efficacy and Safety of an mRNA-Based RSV PreF Vaccine in Older Adults. N Engl J Med **2023**; 389:2233-44.
